# Nebulized bone marrow-derived stem cell supernatant induces tolerogenic dendritic cells via upregulation of FOXO3 for EAE treatment

**DOI:** 10.1186/s12974-025-03690-2

**Published:** 2026-01-14

**Authors:** Junfeng Wu, Zhixin Qiao, Yanping Wang, Sifan Zhang, Jiayu Ji, Xiaoru Ma, Xiyu Zhang, Xin Xiu, Xiujuan Lang, Xijun Liu, Bo Sun, Hulun Li, Yumei Liu

**Affiliations:** 1 Department of Neurobiology, School of Basic Medical Sciences, Harbin Medical University, No. 194 Xuefu Road, Harbin, Heilongjiang 150081 PR China; 2 Key Laboratory of Preservation of Human Genetic Resources and Disease Control in China, Harbin Medical University, Ministry of Education, Harbin, 150081 China; 3 The Key Laboratory of Myocardial Ischemia, Harbin Medical University, Harbin, China

**Keywords:** Stem cell supernatant, Experimental autoimmune encephalomyelitis, Dendritic cell, Antigen presentation, FOXO3

## Abstract

**Background:**

Bone marrow mesenchymal stem cells (BMSCs) exert potent paracrine effects that can reshape the immune microenvironment. Nebulized inhalation enables non-invasive, targeted delivery to the lung, a pivotal immune interface capable of modulating systemic immunity. This study introduces a novel therapeutic strategy using nebulized BMSC supernatant to activate the FOXO3 signaling pathway in pulmonary dendritic cells (DCs), reprogramming them toward a tolerogenic phenotype. This approach suppresses autoreactive T cell infiltration and alleviates central nervous system (CNS) inflammation in the experimental autoimmune encephalomyelitis (EAE) model, offering a potential acellular therapy for multiple sclerosis (MS) and other autoimmune diseases.

**Methods:**

EAE mice were treated with nebulized BMSC supernatant or adoptive transfer of pretreated DCs. Disease progression was assessed by body weight and clinical scores. Hematoxylin and eosin (HE) staining and myelin immunofluorescence staining were used to evaluate CNS inflammation and demyelination. Flow cytometry measured T cell differentiation in spleen and lymph nodes, as well as DC antigen presentation and cytokine secretion in lung tissue. Quantitative Polymerase Chain Reaction (QPCR) assessed inflammatory cytokines, and immunofluorescence determined FOXO3 expression in DCs. FOXO3 involvement was validated using inhibitor-pretreated DCs in adoptive transfer experiments.

**Results:**

Nebulized BMSC supernatant reduced clinical severity and weight loss in EAE mice, and decreased CNS inflammatory infiltration and demyelination. Treatment suppressed peripheral Th1 and Th17 differentiation while increasing Treg frequency. Lung DCs exhibited reduced antigen presentation and pro-inflammatory cytokine expression, increased IL-10 secretion, and elevated FOXO3 expression. Blocking FOXO3 in DCs reversed these effects, aggravating EAE symptoms and promoting Th1/Th17 differentiation.

**Conclusions:**

Nebulized BMSC supernatant effectively treats EAE by inducing pulmonary tolerogenic dendritic cells (tolDC) via FOXO3 activation, reshaping peripheral T cell responses, and reducing CNS inflammation. This strategy highlights the therapeutic potential of targeting the pulmonary immune interface as a novel acellular approach for MS and related autoimmune disorders.

**Graphical Abstract:**

**Figure:**
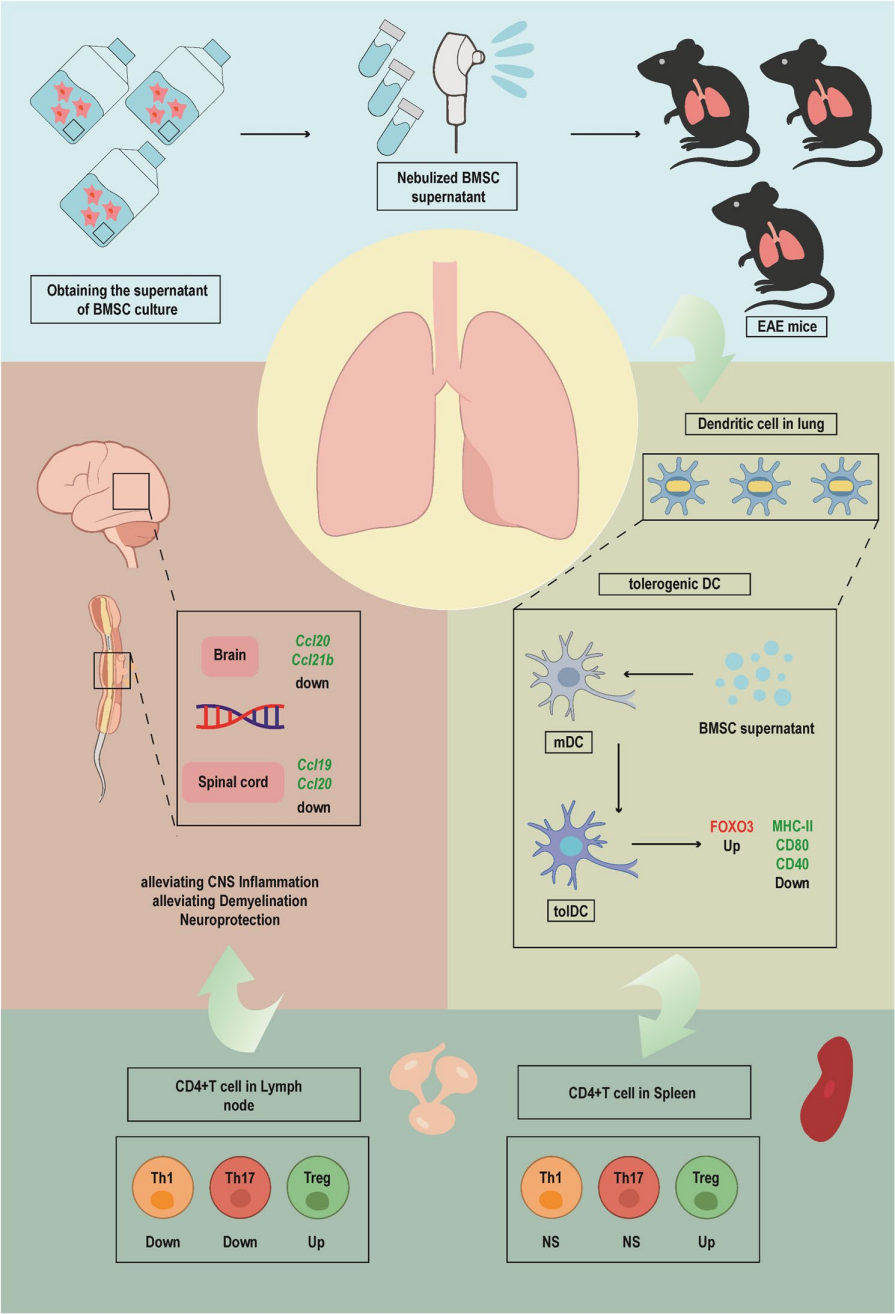
The schematic illustrates our workflow and main findings in that nebulized BMSC supernatant drives DCs in the lungs of EAE mice toward a tolerogenic phenotype, leading to increased FOXO3 expression. This shift modulates immune cell differentiation and the production of multiple cytokines, ultimately attenuating CNS inflammation and enhancing neuroprotection in EAE mice.

**Supplementary Information:**

The online version contains supplementary material available at 10.1186/s12974-025-03690-2.

## Introduction

Multiple sclerosis (MS) is a chronic autoimmune disorder of the central nervous system (CNS) characterized by inflammation, demyelination, and neurodegeneration, leading to progressive neurological disability in young adults worldwide [1–3]. Although current therapies can alleviate symptoms and delay disease progression, they often require long-term systemic administration and carry risks of immunosuppression, highlighting the need for more targeted and less invasive strategies [4].

Experimental autoimmune encephalomyelitis (EAE) is the most widely used animal model of MS, reproducing key pathological features including CNS immune cell infiltration, demyelination, and axonal injury [5–7]. Pathogenesis in both EAE and MS is driven largely by activated Th1 and Th17 cells, which are primed by dendritic cells (DCs) in peripheral tissues before crossing the blood–brain barrier to mediate CNS damage and perpetuate inflammation [8].

Recent evidence suggests that, similar to the gut–brain axis, a bidirectional “lung–brain axis” exists [9]. In EAE, autoreactive T cells can transiently reside in lung tissue, undergo transcriptional reprogramming, and acquire enhanced CNS homing ability [10]. Epidemiological data linking lung infections and smoking to increased MS risk further support the lungs as an immunological gateway to the CNS [11, 12]. Targeting this pulmonary immune interface may therefore provide a novel route to modulate pathogenic immune cell trafficking.

Bone marrow mesenchymal stem cells (BMSCs) possess potent immunomodulatory properties mediated largely by paracrine secretion of bioactive factors, collectively termed the secretome or conditioned medium (CM) [13, 14]. Cell-free MSC-CM therapy offers advantages over cell transplantation, including reduced immunogenicity, longer shelf-life, and lower cost [15]. Previous studies have demonstrated protective effects of MSC-CM in various autoimmune and neuroinflammatory conditions, including cerebral ischemia and EAE [16–18].

Here, we hypothesized that nebulized inhalation of BMSC supernatant could locally reprogram lung DCs toward a tolerogenic phenotype via activation of the transcription factor FOXO3 [19], thereby reshaping peripheral T cell differentiation and attenuating CNS inflammation in EAE. To our knowledge, this is the first study to target the pulmonary immune interface with nebulized MSC-derived factors as a non-invasive therapeutic approach for MS.

## Materials and methods

### Animals

The animal model was established using 6-8-week-old female C57BL/6J mice obtained from Beijing Huafu Kang Co (Beijing, China). Throughout the experiment, the mice had unrestricted access to food and were housed in an animal facility maintained at a temperature of approximately 24 °C, with a humidity level around 55%, under a 12-hour light/dark cycle, and kept in specific pathogen free (SPF) conditions. All animal experiments were conducted following the guidelines for the care and use of experimental animals established by the Chinese National Institute of Health, and the experimental protocols were approved by the Ethics Committee of Harbin Medical University.

### Extraction of BMSC supernatant

Bone marrow mesenchymal stem cells (purchased from Cyagen, Guangzhou, China) from C57BL/6 mice were cultured in culture flasks until they reached 80%-90% confluence. After digestion and counting, the cells were transferred to T25 culture flasks at a density of 2.5 × 10^6^ cells/cm². Twelve hours post-seeding, the cells were washed twice with sterile PBS. The medium was then replaced with serum-free DMEM, and BMSCs were further cultured for 48 h before collecting BMSC supernatant.

### EAE induction and therapy

The recombinant mouse myelin oligodendrocyte glycoprotein (rMOG) was mixed with CFA in equal volumes and vortexed to create a water-in-oil emulsion. Mice were subjected to multiple subcutaneous injections of 100 µg of this emulsion on the back, and Pertussis Toxin (PTX, 200 ng/mouse, List Biological Laboratories, 180, USA) solution was injected into the tail vein on the day of immunization (day 0) and the following day after immunization. Mice in the nebulized supernatant group and the nebulized DMEM medium group were treated with a compressed nebulizer (PARI, JuniorBoy3305) for 30 min daily starting from day 7 post-immunization, using 5 mL of either BMSC supernatant or serum-free DMEM medium. As a positive control, mice in the tail vein supernatant group received a tail vein injection of 50 µL of BMSC supernatant every two days. Body weight and clinical scores were recorded daily beginning on the day of immunization.

The scoring criteria were as follows [20, 21]: 0, Asymptomatic; 0.5, Tail tip paralysis; 1, Total tail paralysis; 2, Abnormal gait; 2.5, Unilateral hindlimb paralysis; 3, Bilateral hindlimb paralysis; 3.5, Unilateral hindlimb paralysis with paralysis of lateral forelimbs; 4, Quadriplegia; 5, Death.

### Adoptive transfer of dendritic cells

After euthanizing the mice, the femurs and tibias were extracted, and the bone marrow cells were flushed out. Following the lysis of red blood cells with ACK (Ammonium-Chloride-Potassium) buffer, the cells were cultured in RPMI 1640 complete medium containing 10% FBS, recombinant mouse GM-CSF (20 ng/mL, Abbkine, PRP2116, China), and IL-4 (10 ng/mL, Abbkine, PRP1137, China) at 37℃ in a 5% CO_2_ incubator to obtain immature dendritic cells. On day 9, the control group was treated with serum-free DMEM containing LPS (1 µg/mL, Meilunbio, MB5198-T, China), the supernatant group received supernatant containing LPS, and the inhibitor group was given supernatant containing LPS and Carbenoxolone (100 µM, MCE, HY-B1367, USA). After an additional 2 days of culture, the cells were collected. On days 5, 7, 9, and 11 post-EAE immunization, 1 × 10^6^ cells/mouse were injected via the tail vein, resulting in the groups EAE + DC, EAE + DC(Sup), and EAE + DC(Sup + Inh).

### Preparation of mononuclear cells

Mice were euthanized using 5% isoflurane, followed by cardiac perfusion with cold Hank’s Balanced Salt Solution (HBSS) devoid of Mg^2+^ and Ca^2+^. Brain, spinal cord, spleen, lung, and lymph node tissues were collected to prepare a mononuclear cell suspension. The spleen, lung, and lymph nodes were ground and filtered through a 40 μm nylon mesh, and then centrifuged at 500×g for 6 min at 4℃. Red blood cells were lysed using ACK (Ammonium-Chloride-Potassium) buffer. The resulting mononuclear cells were suspended in 1640 medium for subsequent experiments. After grinding and homogenizing the brain and spinal cord tissue, it was centrifuged at 300×g for 10 min at 4℃. The mixture was filtered through a nylon mesh and centrifuged again at 300×g for 10 min. Subsequently, 30% Percoll was added to resuspend the cells, and the mixture was centrifuged at 300×g for 30 min at room temperature without brake application. The myelin layer and supernatant were discarded, and the cells were resuspended in D-HBSS. After a final centrifugation at 300×g for 10 min at 4℃, the supernatant was discarded, and the cells were resuspended in 10% 1640 medium for subsequent experiments.

### Immunofluorescence

Lung tissue was embedded and sectioned, followed by deparaffinization. Antigen retrieval was performed using citrate buffer, and after natural cooling, the sections were treated with 3% H_2_O_2_ to eliminate endogenous peroxidase activity. The sections were then blocked with horse serum containing 0.3% Triton at room temperature for 90 min to reduce nonspecific binding. Primary antibodies FOXO3 (1:500, CellSignaling, 12829, USA) and CD11c (1:500, ThermoFisher, 14-0114-82, USA) were added and incubated overnight at 4℃. The next day, the sections were washed three times with PBS, followed by the addition of Goat anti-Armenian Hamster IgG (H + L) Highly Cross-Adsorbed Secondary Antibody (ThermoFisher, A78963, USA) and Donkey anti-Rabbit IgG (H + L) Highly Cross-Adsorbed Secondary Antibody (ThermoFisher, A-31572, USA), which were incubated at room temperature for 2 h. After washing with PBS again, DAPI was used to stain the nuclei, and the slides were mounted.

### Quantitative real-time PCR (qPCR) analysis

At the peak of EAE, lung tissues from mice were collected, and RNA was extracted using the Trizol (TaKaRa, Tokyo, Japan) method. After reverse transcription into cDNA using M-MLV reverse transcriptase (RNase H) (TaKaRa, 2641B, Japan), DNA amplification was performed with Hieff qPCR SYBR Green Master Mix (High rox plus) (Yeasen, 11203ES08, China), and relative gene expression was calculated using the 2(-Delta Delta) method.

The primer sequences utilized in the experiment were as follows: GAPDH: AGGTCGGTGTGAACGGATTTG (forward) and TGTAGACCATGTAGTTGAGGTCA (reverse); H2-Ab1: AGCCCCATCACTGTGGAGT (forward) and GATGCCGCTCAACATCTTGC (reverse); CD40: TGTCATCTGTGAAAAGGTGGTC (forward) and ACTGGAGCAGCGGTGTTATG (reverse); CD80: ACCCCCAACATAACTGAGTCT (forward) and TTCCAACCAAGAGAAGCGAGG (reverse); Csf2: GGCCTTGGAAGCATGTAGAGG (forward) and GGAGAACTCGTTAGAGACGACTT (reverse); Il10: GCTCTTACTGACTGGCATGAG (forward) and CGCAGCTCTAGGAGCATGTG (reverse); Tnf: GACGTGGAACTGGCAGAAGAG (forward) and TTGGTGGTTTGTGAGTGTGAG (reverse).

### Flow cytometry analysis

Single cells obtained from the lungs, spleen, lymph nodes, and CNS were subjected to cell viability staining (ThermoFisher, 65-0865-18, USA), followed by surface staining with antibodies anti-CD45, anti-CD4, anti-MHC-II, anti-CD80, and anti-CD40 (BioLegend, San Diego, CA, USA). Intracellular staining was performed after membrane permeabilization (BD, 554714, USA) using antibodies anti-GM-CSF, anti-TNF-α, anti-IL-10, anti-IL-17 and anti-IFN-γ (BioLegend, San Diego, CA, USA). Nuclear staining was conducted post-nuclear permeabilization (ThermoFisher, 00-5523-00, USA) with antibodies anti-Foxp3 (ThermoFisher, 17-5773-82, USA) and anti-FOXO3 (CellSignaling, 12829, USA). Surface staining of stem cells was carried out using antibodies anti-CD29, anti-CD31, anti-CD34, anti-CD44, anti-SCA-1, and anti-CD117 (BioLegend, San Diego, CA, USA). The samples were then incubated at 4℃ for 30 min, washed with PBS, and analyzed using a FACS Verse flow cytometer, with data processed using FlowJo software.

### Identification of adipogenic, osteogenic and chondrogenic differentiation of BMSCs

BMSCs were seeded at a density of 2 × 10^4^ cells/cm² in six-well plates coated with 0.1% gelatin. When the cell confluence reached approximately 90%, mesenchymal stem cell adipogenic induction medium (Cyagen, MUXMX-90031, USA) was added until the lipid droplets reached the desired size and shape. When the cell confluence was about 70%, mesenchymal stem cell osteogenic induction medium (Cyagen, MUXMX-90021, USA) was added, and after successful induction, Alizarin Red staining was performed. For chondrogenic differentiation, approximately 3 ~ 4 × 10^5^ cells were centrifuged and then exposed to chondrogenic differentiation premix (Cyagen, MUXMX-90041, USA). After washing, chondrogenic induction complete medium (Cyagen, MUXMX-90041, USA) was added until cartilage pellets with a diameter of 1.5–2 mm were formed for subsequent staining.

### Histology staining

In HE staining, after equilibrating the spinal cord sections at room temperature, the tissues were immersed in pathological PBS for 5 min, followed by fixation in cold acetone at 4 ℃ for 15 min. The sections were then washed with pathological PBS for 5 min, repeated three times. After staining with hematoxylin (SOLARBIO, G1120, China) for 3 min, the excess hematoxylin on the tissue sections was rinsed off with running water. The sections were stained with eosin (SOLARBIO, G1120, China) for 3 min, followed by a 7-minute rinse with running water to allow the cell nuclei to return to blue. The slides were then dried, followed by dehydration through an ethanol gradient. After clearing with xylene, the slides were mounted. In Myelin immunofluorescence staining, the sections were incubated in PBS diluted FluoroMyelin Green stains (Molecular Probes, F34651, USA) for 20 min in the dark. The slices were then washed with pathological PBS for 10 min, repeated three times, and subsequently mounted with antifade mounting medium (Abbkine, BMU104-CN, China).

### Statistical analysis

The experimental data were analyzed and plotted by GraphPadPrism9, immunofluorescence images were analyzed by Image J, clinical score and body weight score were analyzed by *two-way ANOVA*, other results were analyzed by *one-way-ANOVA* between the four groups (*P < 0.05, **P < 0.01, ***P < 0.001, ****P < 0.0001).

## Results

### Nebulized BMSC supernatant alleviates CNS inflammation and demyelination

**Fig. 1:**
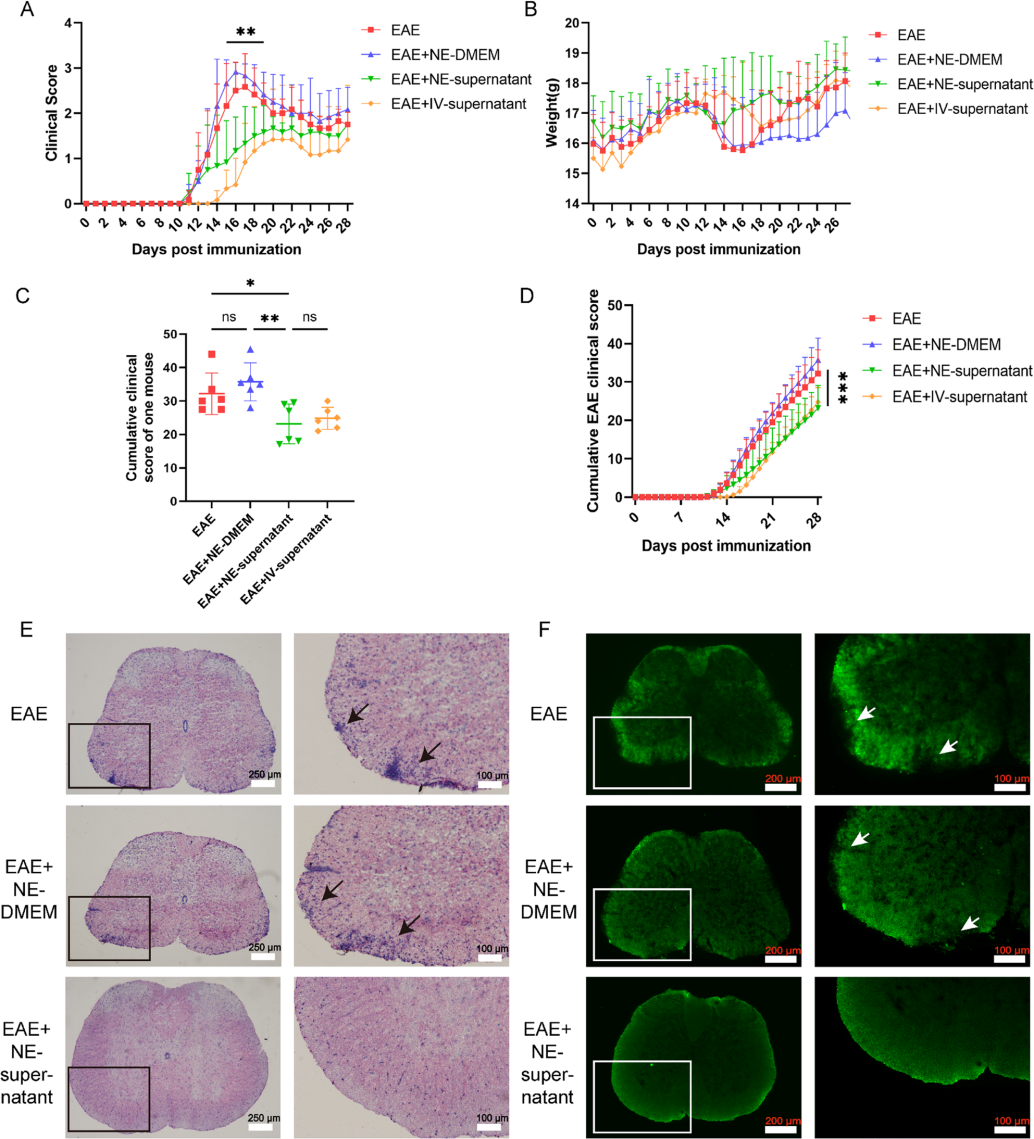
Nebulized BMSC supernatant alleviates CNS inflammation and demyelination. **A** The clinical scores of EAE, EAE + NE-DMEM, EAE + NE-supernatant, and EAE + IV-supernatant group (n = 6). **Significant difference between EAE and EAE + NE-supernatant. **B** The body weight evaluation of EAE, EAE + NE-DMEM, EAE + NE-supernatant, and EAE + IV-supernatant group (n = 6). **A**-**B** Data are expressed as Mean ± SD **p < 0.01, two-way ANOVA. **C** Cumulative clinical scores for each mouse at 28 days (n = 6). **D** Cumulative clinical scores for each mouse per day (n = 6). ***Significant difference between EAE and EAE + NE-supernatant. **C**-**D** Data are expressed as Mean ± SEM, *p < 0.05, **p < 0.01, ***p < 0.001, two-way ANOVA. **E** HE staining of the spinal cord at the peak stage of disease. **F** Myelin immunofluorescence staining of the spinal cord at the peak stage of disease

To validate the therapeutic potential of BMSC supernatant, a positive control group (EAE + IV-supernatant) received tail vein administration. Clinical scores and body weights of mice in each group were monitored over a period of 28 days. Results demonstrated a significant reduction in clinical scores in the EAE + NE-supernatant group compared to the EAE group between the 15th and 19th day, despite a declining trend in scores from the 13th day onwards. Onset of symptoms in the EAE + NE-supernatant group coincided with the EAE and EAE + NE-DMEM on the 11th day, whereas the EAE + IV-supernatant group exhibited symptom onset on the 14th day with the lowest disease severity (Fig. 1A). The cumulative disease scores of the EAE + NE-supernatant and EAE + IV-supernatant groups were significantly lower than those of the EAE and EAE + NE-DMEM groups (Fig. 1C, D). Body weight differences among the groups were not statistically significant (Fig. 1B). Moreover, the nebulized BMSC supernatant also reduced the clinical scores in the Mog_35 − 55_-induced EAE mouse model. Additionally, nebulized human umbilical cord mesenchymal stem cells (hucMSC) supernatant achieved the same therapeutic effect in the rMog-induced EAE model (Fig. S3A-D). These findings suggest that nebulized supernatant effectively reduces EAE clinical scores, achieving comparable therapeutic outcomes to tail vein supernatant. Furthermore, there were no notable differences in clinical scores between the nebulized DMEM and EAE groups.

The therapeutic efficacy of nebulized BMSC supernatant was validated through histological analysis using HE staining and immunofluorescence staining of spinal cord myelin. The findings indicated a marked reduction in inflammatory infiltration in the spinal cord during the peak phase in the EAE + NE-supernatant group compared to the EAE group and the EAE + NE-DMEM group (Fig. 1E; Fig. S3E). Furthermore, immunofluorescence staining of spinal cord myelin demonstrated a significant decrease in demyelination levels in the EAE + NE-supernatant group (Fig. 1F; Fig. S3F). Collectively, these results demonstrate that nebulized BMSC supernatant effectively reduces EAE severity, suppresses CNS inflammation, and preserves myelin integrity, achieving efficacy comparable to intravenous delivery.

### Nebulized supernatant reduces the infiltration of antigen-specific T and B cells in the CNS of EAE mice

**Fig. 2:**
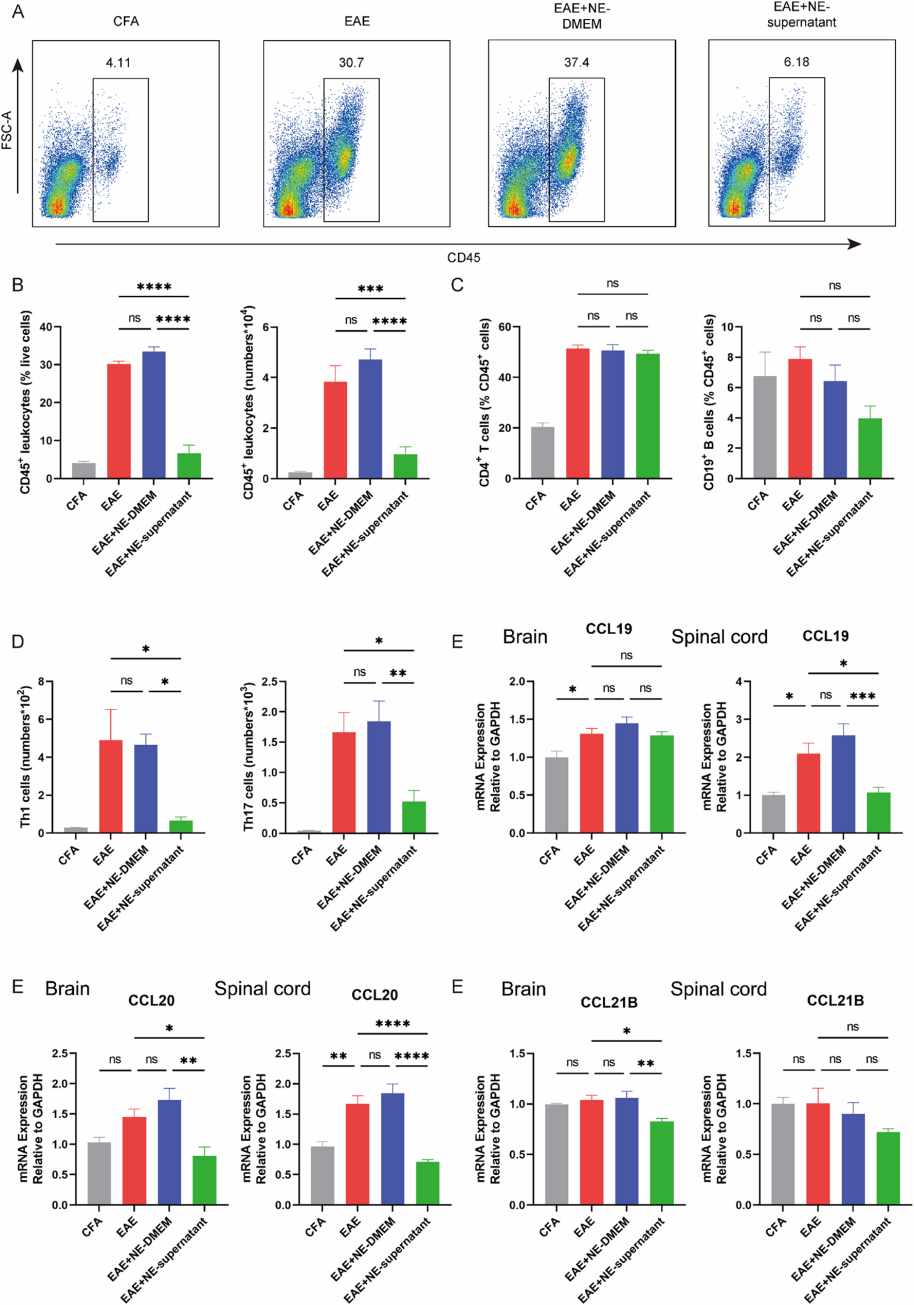
Nebulized BMSC supernatant alleviates CNS inflammation in EAE mice. **A** Representative flow cytometry plots of CD45+ leukocytes from mice CNS at the peak stage of disease. **B** The percentages and numbers of CD45+ leukocytes were determined by flow cytometry. **C** The percentages of CD4+ T cells and CD19+ B cells were determined by flow cytometry. **D** The numbers of Th1 and Th17 cells were determined by flow cytometry. **E** The mRNA expression levels of Ccl19 in brain and spinal cord. **F** The mRNA expression levels of Ccl20 in brain and spinal cord. **G** The mRNA expression levels of Ccl21b in brain and spinal cord. **B**-**G** Data are expressed as Mean ± SEM, *p < 0.05, **p < 0.01, ***p < 0.001, ****p < 0.0001, one-way-ANOVA

Further flow cytometry staining of the CNS was conducted, and the results were consistent with the tissue staining results. After nebulization, both the percentage and the number of CD45^+^ leukocytes significantly decreased after nebulization (Fig. 2A, B). Although there was no significant change in the proportions of CD4^+^ T cells and CD19^+^ B cells following the nebulization of the supernatant, the number of antigen-related (CD4^+^ IFN-γ^+^) Th1 and (CD4^+^ IL-17^+^) Th17 cells significantly decreased (Fig. 2C, D; Fig. S3G, H). Correspondingly, the expression of the chemokines CCL19, CCL20, and CCL21 in the brain and spinal cord was also reduced (Fig. 2E-G). The results indicate that the nebulized supernatant can influence the transport of antigen-specific Th1 and Th17 cells to the CNS.

### Nebulized supernatant modulates peripheral T cell polarization

**Fig. 3:**
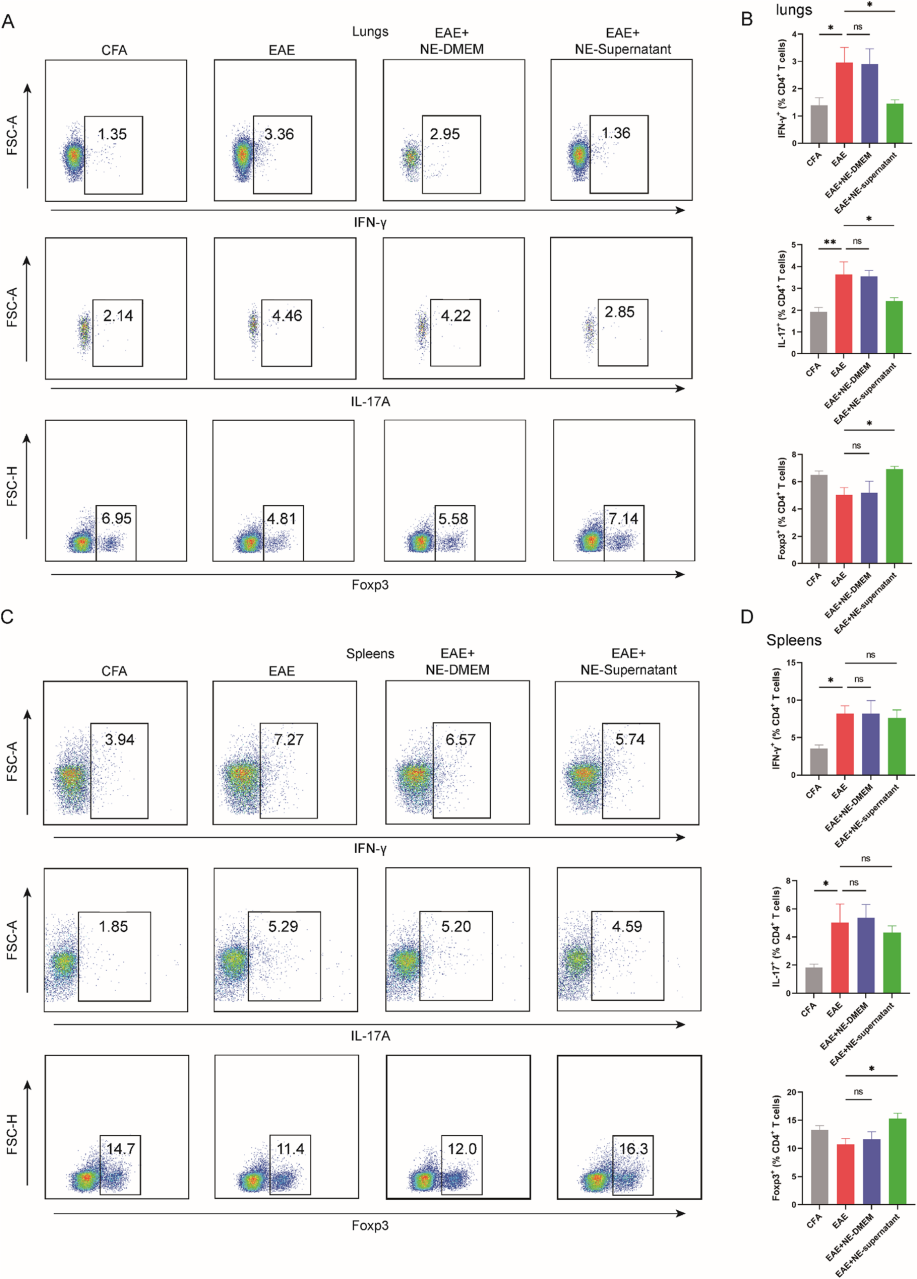
Nebulized supernatant modulates peripheral T cell polarization. **A** Representative flow cytometry plots of CD4+ T cells expressing IFN-γ, IL-17 and Foxp3 in lung at the peak stage of disease. **B** Representative flow cytometry plots of CD4+ T cells expressing IFN-γ, IL-17 and Foxp3 in spleen at the peak stage of disease. **C** The percentages of CD4+ T cells expressing IFN-γ, IL-17 and Foxp3 in lung at the peak stage of disease (n = 6). **D** The percentages of CD4+ T cells expressing IFN-γ, IL-17 and Foxp3 in spleen at the peak stage of disease (n = 5). **C**-**D** Data are expressed as Mean ± SEM, *p < 0.05, **p < 0.01, one-way-ANOVA

Flow cytometry of lung and spleen cells at peak disease revealed that EAE induction increased (CD4^+^ IFN-γ^+^) Th1 and (CD4^+^ IL-17^+^) Th17 frequencies while reducing (CD4^+^ Foxp3^+^) Treg levels in CD4⁺ T cells. Nebulized BMSC supernatant treatment reversed these changes in the lung, significantly decreasing Th1 and Th17 proportions and increasing Treg frequency compared with untreated EAE mice (Fig. 3A, C). In the spleen, nebulized BMSC supernatant significantly increased Treg frequency but did not cause significant changes in Th1 or Th17 levels, although a downward trend was observed (Fig. 3B, D). Similar trends were found in lymph nodes (Fig. S4). Nebulized DMEM had no effect on T cell polarization in any peripheral tissue examined. These results indicate that nebulized BMSC supernatant promotes peripheral immune tolerance by enhancing Treg differentiation and suppressing pro-inflammatory Th1/Th17 responses, particularly in the pulmonary compartment.

### Nebulized BMSC supernatant influences antigen presentation and cytokine secretion in lung DCs

QPCR analysis showed that nebulized BMSC supernatant significantly reduced pro-inflammatory cytokine transcripts (*Csf2*, *Tnf*, *Ifng*) and antigen presentation–related genes (*H2-Ab1*, *Cd40*, *Cd80*), while increasing *Il4* and trending toward higher *Il10* and *Il33* expression in lung tissue (Fig. S6A-D).

**Fig. 4:**
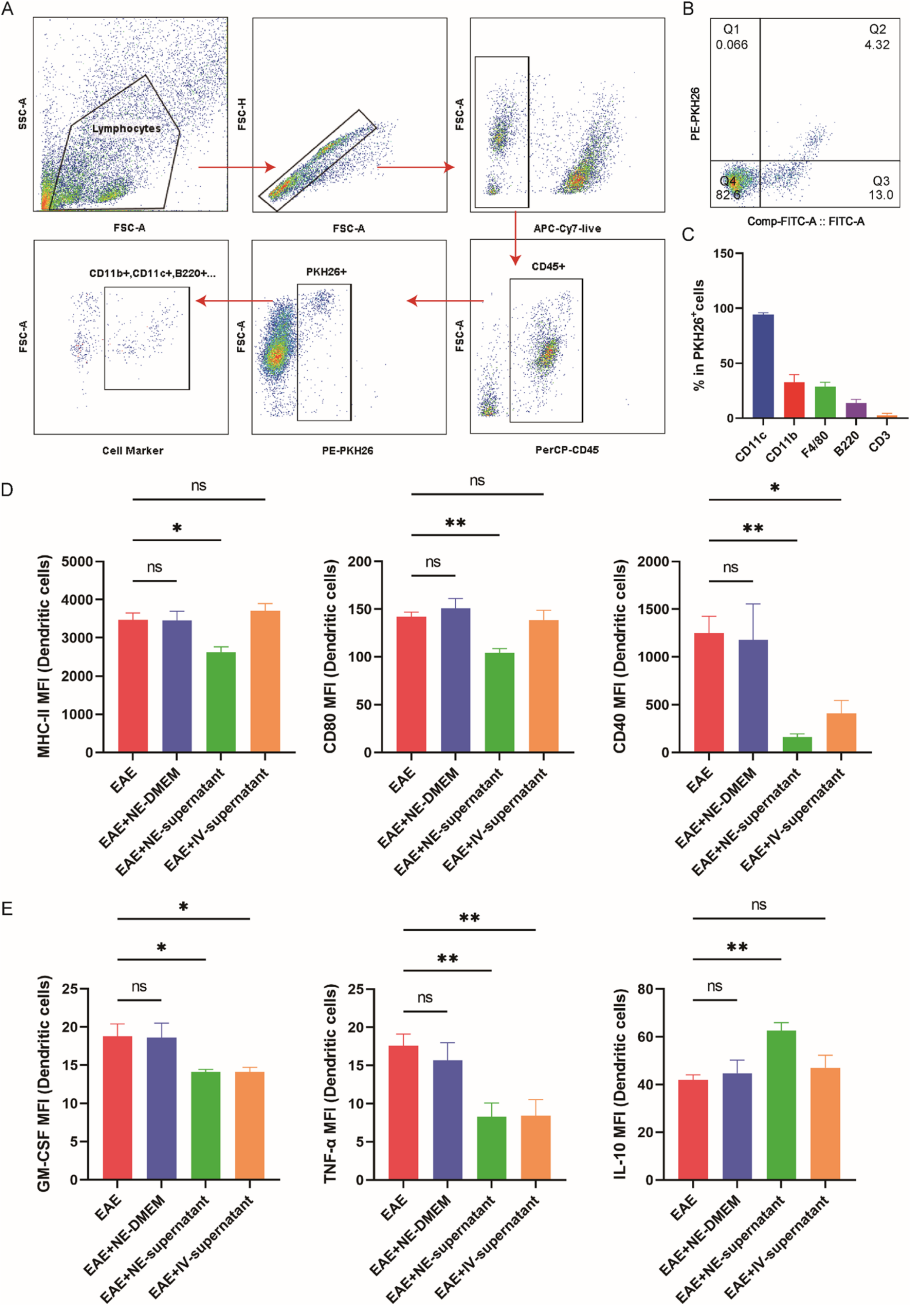
Nebulized BMSC supernatant influences antigen presentation and cytokine secretion in lung DCs. **A** The gate strategy for analysis of exosomes uptake by specific cells in the lung. **B** Detection of exosomes uptake by CD11c+ DCs. **C** Flow cytometric detection of exosomes uptake by cells in the lung (n = 5). **D** Mean fluorescent intensity (MFI) of MHC-II, CD80, and CD40 expression by lung CD11c+ DCs (n = 5). **E** Mean fluorescent intensity of GM-CSF, TNF-α, and IL-10 expression by lung CD11c+ DCs (n = 5). **D**-**E** Data are expressed as Mean ± SEM, *p < 0.05, **p < 0.01, one-way-ANOVA

These results suggest that nebulized BMSC supernatant alleviated inflammation in lung tissue and remodeled the lung immune microenvironment. To further investigate the uptake and distribution of nebulized supernatant within lung tissue, PKH26 was employed to label the exosomal components in the supernatant. PKH26 tracing demonstrated that exosomal components of the nebulized supernatant were preferentially taken up by CD11c⁺ DCs, which represented the predominant PKH26⁺ population, exceeding CD11b⁺ monocytes/neutrophils, F4/80⁺ macrophages, B220⁺ B cells, and CD3⁺ T cells (Fig. 4A-C). Phenotypic and functional analysis revealed that DCs from the EAE + NE-supernatant group exhibited reduced MHC-II, CD80, and CD40 expression, decreased GM-CSF and TNF production, and elevated IL-10 secretion (Fig. 4D, E), consistent with a tolerogenic phenotype. Notably, intravenous supernatant did not alter these DC markers, suggesting a lung-specific mechanism for inhalation therapy.

### FOXO3 mediates the tolerogenic reprogramming of lung DCs by nebulized BMSC supernatant

**Fig. 5:**
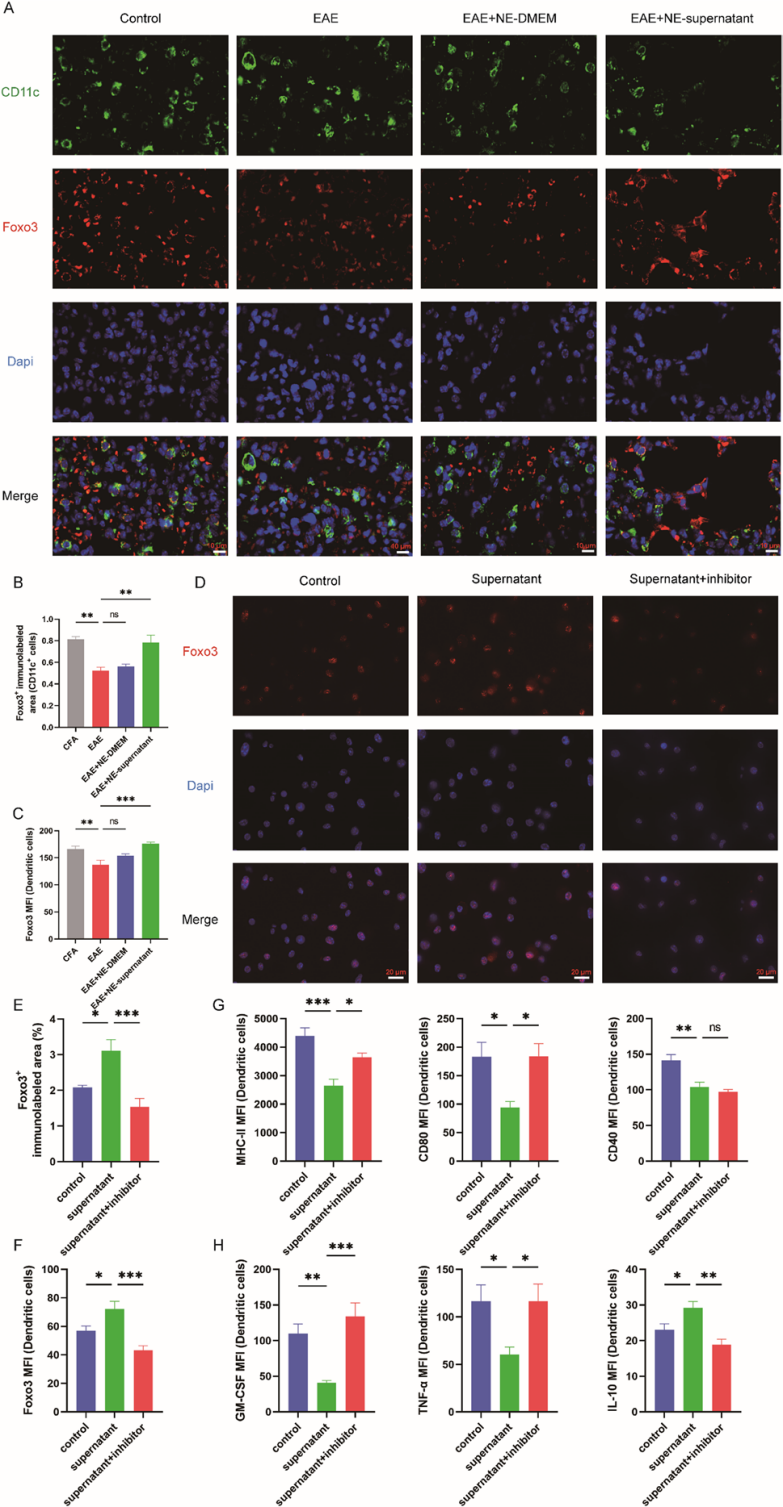
FOXO3 mediates the tolerogenic reprogramming of lung DCs by nebulized BMSC supernatant. **A** Immunofluorescence staining of CD11c and FOXO3 was conducted in the lung tissues of the CFA, EAE, EAE + NE - DMEM, and EAE + NE-supernatant groups. **B** Percentage of the immunolabeled area of FOXO3 in CD11c+ DCs (n = 4). **C** Mean fluorescent intensity (MFI) of FOXO3 expression by lung CD11c+ DCs (n = 5). **D** Immunofluorescence staining of FOXO3 was conducted in the BMDCs of the Control, Supernatant, Supernatant + inhibitor groups. **E** Percentage of the immunolabeled area of FOXO3 in BMDCs (n = 5). (F) Mean fluorescent intensity (MFI) of FOXO3 expression by BMDCs (n = 7). **G** Mean fluorescent intensity (MFI) of MHC-II, CD80, and CD40 expression by BMDCs (n = 5). **H** Mean fluorescent intensity of GM-CSF, TNF-α, and IL-10 expression by BMDCs (n = 5). **B**-**C**, **E**-**H** Data are expressed as Mean ± SEM, *p < 0.05, **p < 0.01, ***p < 0.001, one-way-ANOVA

FOXO3 is a crucial transcription factor that limits the production of inflammatory cytokines by DCs and regulates T cell survival. Therefore, we hypothesize that the nebulized supernatant may mediate the progression of EAE by influencing the differentiation of lung-resident T cells through the modulation of FOXO3. Immunofluorescence analysis revealed that FOXO3 expression in lung DCs of EAE mice at peak disease was significantly reduced compared with the CFA group. Nebulized BMSC supernatant treatment markedly increased FOXO3 expression (Fig. 5A, B). Flow cytometry confirmed the upregulation of FOXO3 in lung DCs following nebulization (Fig. 5C).

To further investigate whether BMSC supernatant influences function by regulating the expression of FOXO3 in DCs, bone marrow-derived dendritic cells (BMDCs) were extracted in vitro (Fig. S7A, B) and cultured under inflammatory conditions with BMSC supernatant. Both immunofluorescence and flow cytometry demonstrated a significant increase in FOXO3 expression. This was accompanied by decreased expression of antigen presentation molecules (MHC-II, CD80, CD40) and pro-inflammatory cytokines (TNF-α, GM-CSF), along with increased IL-10 production, consistent with a tolerogenic DC phenotype, to exclude the influence of other confounding factors, we isolated lung-resident DCs using magnetic nanobeads, and flow cytometry demonstrated an excellent isolation efficiency of over 90%, lung tissue-derived DCs were subjected to the same induction protocol as that used for BMDCs, the results showed that FOXO3 expression in lung tissue DCs was significantly upregulated after treatment with BMSC supernatant, moreover, the phenotypic changes in MHC-II, CD40 and CD80 expression were consistent with those observed in BMDCs, as were the secretion profiles of the cytokines IL-10, TNF-α and GM-CSF. Application of the FOXO3 inhibitor Carbenoxolone (CBX, 100 µM), prevented the supernatant-induced upregulation of FOXO3 (Fig. 5D-F; Fig. S7C-D; Fig. S8). In the presence of CBX, MHC-II, CD80, TNF-α, and GM-CSF expression levels were restored, and IL-10 production returned to control levels (Fig. 5G, H). Collectively, these findings indicate that nebulized BMSC supernatant reprograms lung DCs toward a tolerogenic phenotype by upregulating FOXO3, thereby modulating antigen presentation and cytokine secretion, which may contribute to the attenuation of EAE pathology.

### Adoptive transfer of tolerogenic DCs replicates the therapeutic effect of nebulized BMSC supernatant in a FOXO3-dependent manner

To determine whether the therapeutic effect of nebulized BMSC supernatant is mediated via tolerogenic DCs, we adoptively transferred BMDCs into EAE mice under three conditions: untreated DCs (EAE + DC), BMSC supernatant–pretreated DCs (EAE + DC(Sup)), and BMSC supernatant–pretreated DCs with FOXO3 inhibition (EAE + DC(Sup + Inh)).

**Fig. 6:**
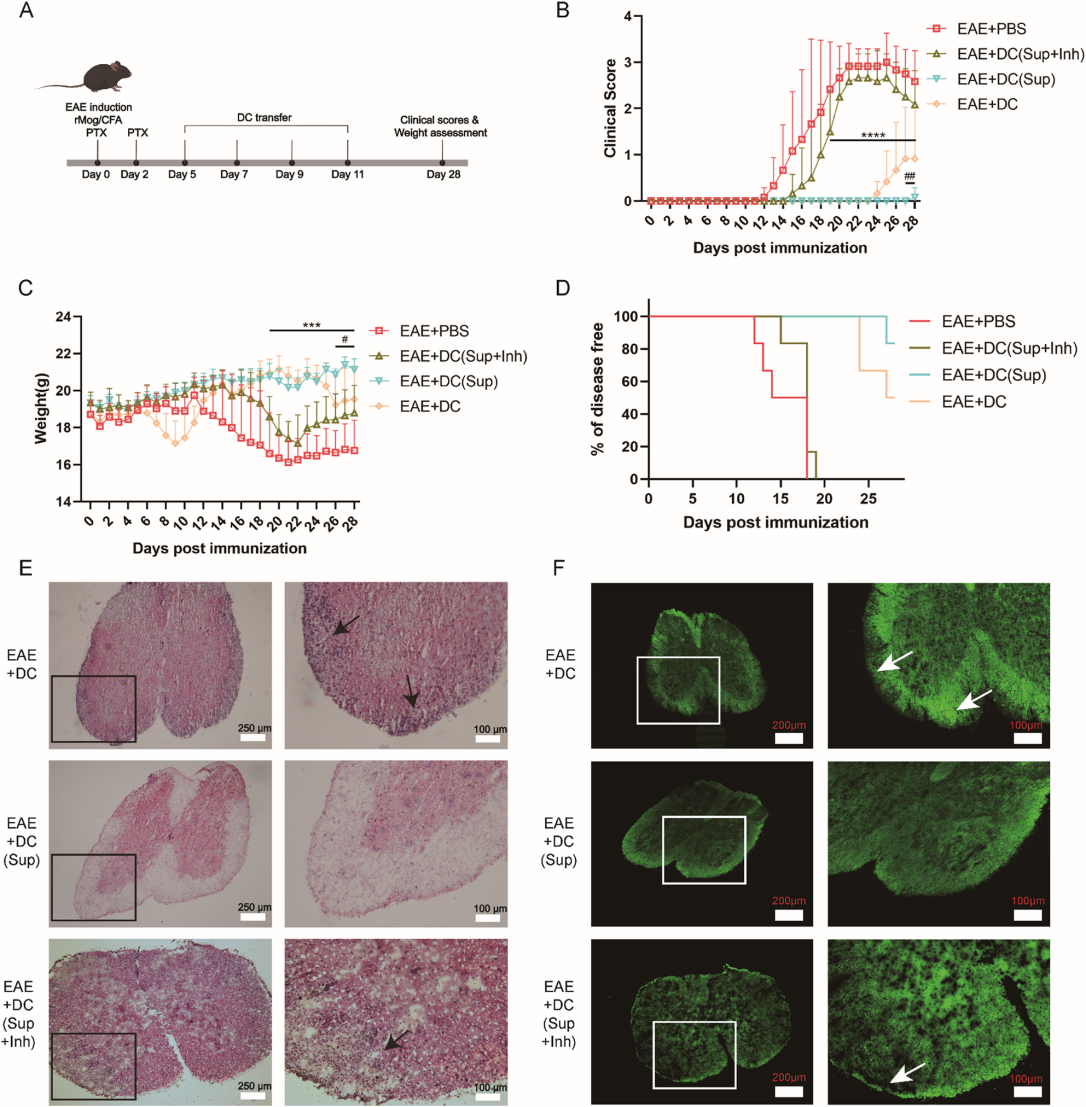
Adoptive transfer of tolerogenic DCs replicates the therapeutic effect of nebulized BMSC supernatant in a FOXO3-dependent manner. **A** Schematic diagram of adoptive transfer of dendritic cells. **B** The clinical scores of EAE, EAE + DC, EAE + DC(Sup), and EAE + DC(Sup + Inh) group (n = 6). ****Significant difference between EAE + DC(Sup) and EAE + DC(Sup + Inh). ##Significant difference between EAE + DC and EAE + DC(Sup). **C** The body weight evaluation of EAE, EAE + DC, EAE + DC(Sup), and EAE + DC(Sup + Inh) group (n = 6). **B**-**C** Data are expressed as Mean ± SD, **p < 0.01, two-way ANOVA. **D** Percentage of disease-free animals (n = 6). **E** HE staining of the spinal cord on the 28th day post-immunization. **F** Myelin immunofluorescence staining of the spinal cord on the 28th day post-immunization

In the EAE + DC group, disease onset was delayed and clinical scores were reduced compared with the EAE group, consistent with previous reports. Notably, EAE + DC(Sup) mice exhibited a further delay in onset, in some cases with almost complete prevention of clinical symptoms, and showed a markedly reduced incidence and significantly lower clinical scores throughout the observation period (*p* < 0.05 vs. EAE + DC). In contrast, inhibition of FOXO3 in supernatant-pretreated DCs abolished these benefits, with the EAE + DC(Sup + Inh) group displaying early onset and high clinical scores comparable to untreated EAE mice. Similar trends were observed in body weight monitoring (Fig. 6A-D). HE staining and myelin immunofluorescence at day 28 revealed reduced inflammatory cell infiltration and demyelination in the EAE + DC(Sup) group compared with both the EAE + DC and EAE + DC(Sup + Inh) groups. Pathological scores were lowest in the EAE + DC(Sup) group (Fig. 6E-F).

These results demonstrate that adoptive transfer of tolerogenic DCs, generated by BMSC supernatant pretreatment, can effectively mitigate EAE onset and severity. Furthermore, the loss of therapeutic benefit following FOXO3 inhibition confirms that this effect is FOXO3-dependent.

### BMSC supernatant–pretreated DCs modulate peripheral T cell differentiation via a FOXO3-dependent mechanism

**Fig. 7:**
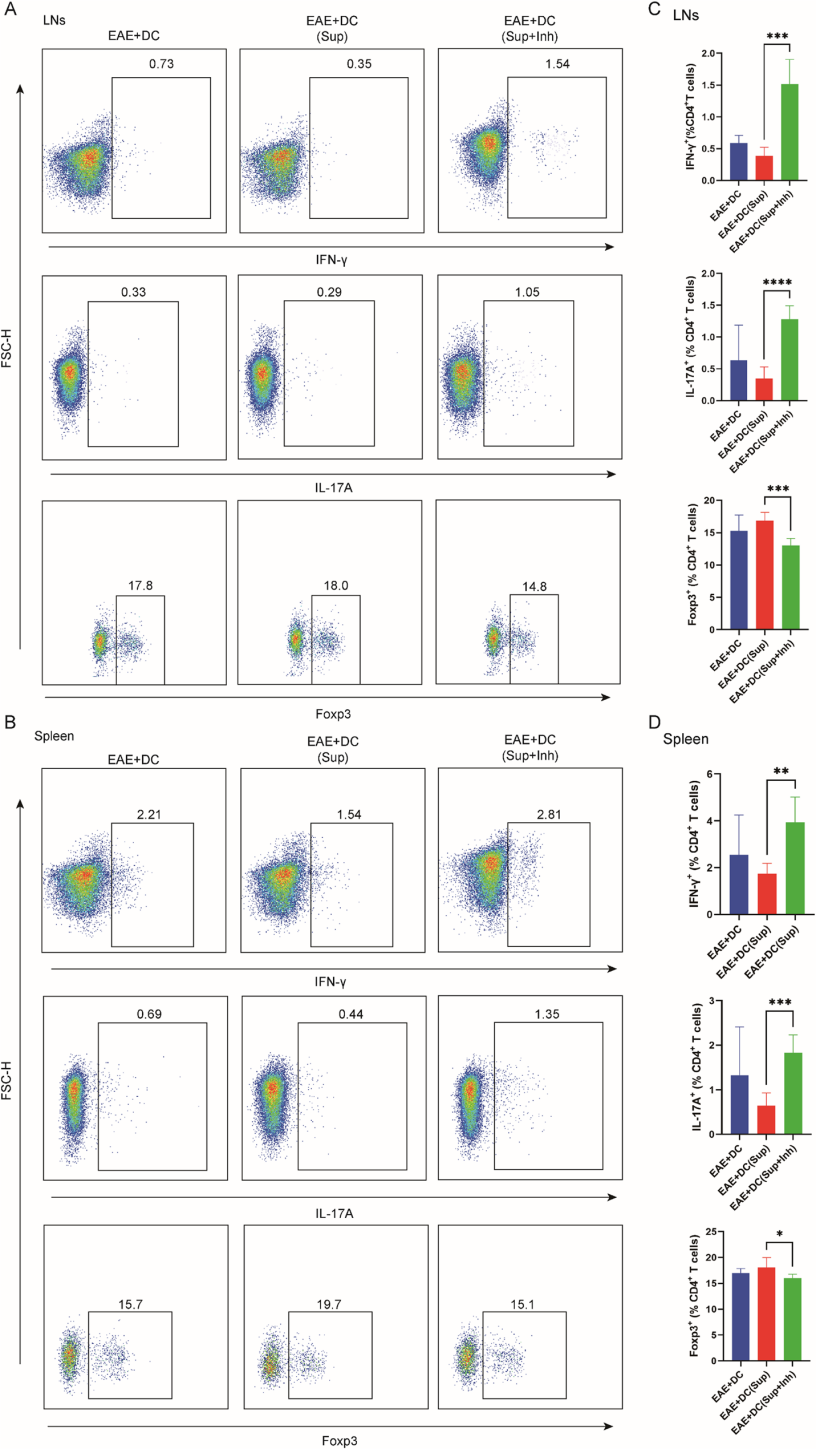
The adoptive transfer of dendritic cells preconditioned with stem cells influences T cell differentiation in peripheral tissues. **A** Representative flow cytometry plots of CD4+ T cells expressing IFN-γ, IL-17 and Foxp3 in lymph node of EAE + DC, EAE + DC(Sup), and EAE + DC(Sup + Inh) group on the 28th day post-immunization. **B** Representative flow cytometry plots of CD4+ T cells expressing IFN-γ, IL-17 and Foxp3 in spleen of EAE + DC, EAE + DC(Sup), and EAE + DC(Sup + Inh) group on the 28th day post-immunization. **C** The percentages of CD4+ T cells expressing IFN-γ, IL-17 and Foxp3 in lymph node of EAE + DC, EAE + DC(Sup), and EAE + DC(Sup + Inh) group on the 28th day post-immunization (n = 5). **D** The percentages of CD4+ T cells expressing IFN-γ, IL-17 and Foxp3 in spleen of EAE + DC, EAE + DC(Sup), and EAE + DC(Sup + Inh) group on the 28th day post-immunization (n = 5). **C**-**D** Data are expressed as Mean ± SEM, *p < 0.05, **p < 0.01, ****p < 0.0001, one-way-ANOVA

Flow cytometry was used to analyze CD4⁺ T cell polarization in the spleen and lymph nodes following adoptive transfer of DCs. Compared with the EAE + DC group, the EAE + DC(Sup) group showed significantly reduced proportions of Th1 (IFN-γ⁺) and Th17 (IL-17⁺) cells, accompanied by an upward trend in Treg frequencies in both tissues. In contrast, when FOXO3 expression was inhibited in the supernatant-pretreated DCs before transfer (EAE + DC(Sup + Inh)), Th1 and Th17 proportions increased markedly, while Treg levels decreased significantly compared with the EAE + DC(Sup) group (Fig. 7A-D). In vitro, the inhibition of FOXO3 can also counteract the differentiation of T cells into Treg induced by the co-culture of supernatant-pretreated BMDC and splenocytes, promoting the differentiation of T cells into Th1 and Th17 subsets (Fig. S10). These findings indicate that supernatant-pretreated DCs can shift peripheral T cell polarization toward an anti-inflammatory profile, thereby potentially suppressing neuroinflammation. The loss of this effect upon FOXO3 inhibition suggests that the mechanism is mediated through FOXO3-dependent modulation of DC function.

## Discussion

MS is a chronic autoimmune disease that affects the CNS and is characterized by immune-mediated inflammation leading to demyelination and axonal damage [22]. It affects approximately 2.3 million people worldwide, primarily impacting young adults aged 18 to 40. Furthermore, the incidence and prevalence of the disease continue to rise globally, with widespread prevalence in North America and Europe. Relapsing-remitting MS is the most common type of MS, diagnosed based on clinical symptoms such as motor weakness, sensory deficits, and vision loss that are not caused by persistent infections or metabolic imbalances. Its occurrence is disseminated in time and space, manifesting as intermittent episodes. The etiology of MS, apart from genetic and environmental factors, may also involve vitamin D deficiency, Epstein-Barr virus infection, obesity, and smoking, which could mediate its onset [23]. Its typical pathological feature is perivenous inflammatory lesions, which include the infiltration of T cells and B cells. These inflammatory processes target myelin antigens, leading to axonal demyelination and neurodegeneration, thereby impairing signal conduction and causing loss of neurological function. The current treatment for MS is a multidisciplinary approach that primarily comprises disease-modifying therapies (DMT), acute relapse treatment, and symptomatic management [24, 25].

EAE is the most commonly used experimental model for MS. Given that the majority of MS patients are young women, female mice aged 6–8 weeks are typically selected to establish the EAE model through active immunization with antigens such as myelin oligodendrocyte glycoprotein (MOG) and proteolipid protein (PLP) [26–28]. Activated Th1 cells and Th17 cells are recognized as key pathogenic cells in both EAE and MS. These cells are initiated by DCs located outside the CNS, subsequently crossing the blood-brain barrier to produce inflammatory products and cytokines that damage myelin and axons. Additionally, they activate resident microglia, which attract more inflammatory cells to the CNS, thereby sustaining the inflammatory cascade [8].

The lungs are vital organs in animals, playing a crucial role in supplying oxygen to tissues. As the organ with the highest oxygen consumption in the human body, the brain’s functions are particularly susceptible to the effects of lung diseases. For instance, pneumonia can exacerbate brain damage following a stroke [29]. Similar to the gut-brain axis, there exists a complex communication network between the CNS and the lungs, referred to as the “lung-brain axis.” Its neuroanatomical basis primarily comprises the vagus nerve and the sympathetic nerves of the upper thoracic spinal cord [30]. Furthermore, there is a significant connection between the lung-brain axis and the endocrine system. In LPS-induced chronic lung infection rat models, inflammatory factors such as lung matrix metalloproteinase (MMP-9) and TNF-α can infiltrate the CNS through various mechanisms, activating glial cells and exacerbating neuronal cell death [31, 32]. Another study indicates that following acute lung injury, elevated levels of systemic cytokines and neutrophils can lead to multiple organ dysfunction, including that of the brain [33]. Additionally, the cytokine storm may represent one of the significant triggers for CNS complications following novel coronavirus infection [34].

Although previous studies suggested that healthy lungs are sterile, recent research has indicated the presence of diverse microbial communities in the lungs [12, 35]. Beyond their role in regulating pulmonary inflammation and the local microenvironment, these microbes may also contribute to CNS disorders. For instance, chronic obstructive pulmonary disease can alter respiratory microflora and increase the risk of Parkinson’s disease (PD) and Alzheimer’s disease (AD) [35]. Furthermore, multiple studies have observed manifestations of respiratory dysfunction due to respiratory muscle weakness in patients with MS. Concurrently, respiratory diseases are a leading cause of morbidity and mortality in MS [36, 37]. In the Lewis rat model of EAE, Odoardi et al. first demonstrated that T cells transiently reside in lung tissue and reprogram their gene expression profiles. Through local proliferation in the lungs and upregulation of adhesion molecule expression, these T cells acquire the ability to enter the CNS. This study supports the view that the migration of T cells to bronchial structures and bronchus-associated lymphoid tissue (BALT) is driven by CCL19 and CCL21 [10]. This may be the mechanism by which respiratory inflammation triggers the relapse of MS. Conditional knockout of ATG7 expression in pulmonary myeloid cells leads to mild inflammation in the lungs and high expression of CCL20, causing the accumulation of Th17 cells in the lungs and significantly delaying the onset of EAE in mice [38, 39]. Another significant finding is that the pulmonary microbiota can modulate the immune reactivity of central nervous tissues. Studies have demonstrated that intrapulmonary perfusion of neomycin can induce a shift in microglia toward the type I interferon signaling pathway, thereby limiting the progression of MS [12]. These studies confirm the potential role of the lung-brain axis in EAE. Additionally, the lungs may serve as an important target for regulating the migration of T cells to autoimmune sites. Compared to invasive intravenous administration, the non-invasive method of specifically targeting the lungs through nebulized drug delivery may offer a novel approach for treating inflammation of the CNS.

MSCs possess potent immunomodulatory and tissue regenerative capabilities. Extensive research has demonstrated that MSCs exert therapeutic effects through paracrine and autocrine mechanisms by producing a range of protective bioactive factors, including immunomodulation, anti-inflammatory activity, and tissue repair [13]. These factors are collectively referred to as the secretome or CM, which primarily encompasses cytokines, chemokines, cell adhesion molecules, lipid mediators, interleukins, growth factors, hormones, exosomes, and microvesicles [14]. As a cell-free technology, MSC-CM transplantation is more convenient and safer than direct MSC transplantation, demonstrating significant potential for clinical translation. Compared to cell-based applications, MSC-CM avoids the risk of host immune reactions, can be stored long-term without the use of toxic cryopreservatives such as DMSO, and is less costly to produce [15]. Research has confirmed that stem cell supernatant not only facilitates wound healing and tissue regeneration but also exerts therapeutic effects in autoimmune diseases such as cerebral ischemia and EAE [16–18, 40].

The supernatant of stem cells can be administered via nebulized inhalation, effectively delivering soluble proteins and vesicular substances with a particle size of 1–3 micrometers to the deep lung tissue. This method of administration is widely utilized in the treatment of lung-related diseases, as it facilitates the delivery of cell vesicles to the lungs through a vibrating mesh nebulizer, thereby alleviating lipopolysaccharide-induced lung injury in mice [41, 42]. Furthermore, clinical studies have confirmed that nebulized exosomes derived from umbilical cord mesenchymal stem cells can treat COVID-19 pneumonia [43]. Compared to intravenous injection, nebulized inhalation is a non-invasive and trauma-free method. This approach not only avoids pain and intestinal side effects but also allows a lower dose of nebulized inhalation to achieve efficacy comparable to that of intravenous injection. Additionally, the nebulized stem cell supernatant retains its antibacterial properties [44]. Selecting an appropriate nebulizer is crucial for maintaining the structural stability of the supernatant components, particularly the cell vesicle structures. Studies indicate that vibrating mesh nebulizers and jet nebulizers cause minimal damage to cell vesicles [41, 45], whereas ultrasonic nebulizers generate heat during operation, leading to the fragmentation of these vesicles. Therefore, vibrating mesh nebulizers and jet nebulizers should be prioritized for nebulizing stem cell supernatant [46].

Our experimental results confirm that nebulized BMSC supernatant can indeed accumulate in the lungs of mice and effectively alleviates CNS inflammation and demyelination in EAE mice, while lowering the number of CNS-infiltrating Th1 and Th17 cells. This supports the concept that targeting the lung immune interface can influence CNS immune activity through the lung-brain axis. Second, we observed that nebulized supernatant modulates peripheral T cell polarization. In lung tissue, there was a marked reduction in Th1 and Th17 cells and an increase in Treg populations, suggesting a shift toward an anti-inflammatory immune profile. Similar, albeit less pronounced, changes were seen in the spleen and lymph nodes. These data are in line with previous findings that altering peripheral T cell balance is critical for suppressing neuroinflammation, and they indicate that the lung serves as an important site for immune reprogramming in EAE.

DCs, macrophages, B cells, and microglia are the classical antigen-presenting cells (APCs). Among these, microglia are regarded as the primary APC population in the CNS, while DCs are recognized as the most effective antigen-presenting cells in the periphery. DCs originate from hematopoietic progenitor cells in the bone marrow, and resident DC subsets are found in the skin, gastrointestinal tract, respiratory tract, and lymphoid organs. These cells are essential for initiating primary adaptive immune responses by capturing and processing antigens, then presenting them to naive CD4^+^ T cells. Activated DCs express MHC-II molecules, co-stimulatory receptors (such as CD80, CD40, and CD86), and pro-inflammatory cytokines (including TNF-α, GM-CSF, and IL-6), which induce T cell proliferation and differentiation into Th1 and Th17 cells [47]. In contrast to classical immunogenic DCs, tolerogenic DCs (tolDCs) typically display lower levels of surface co-stimulatory molecules and higher levels of anti-inflammatory cytokines (such as IL-10 and TGF-β). These characteristics promote Treg differentiation, induce T cell anergy or apoptosis, maintain immune tolerance and homeostasis, and inhibit autoimmune responses [48–50]. TolDC-based therapies and in vivo DC-targeting approaches have been widely applied in clinical trials for the treatment of autoimmune diseases, including type 1 diabetes, rheumatoid arthritis, MS, and Crohn’s disease [49]. In EAE, the expression levels of MHC-II, co-stimulatory molecules, and various T cell polarization factors in microglia are significantly elevated. However, increasing evidence suggests that DCs, the primary antigen-presenting cells in the peripheral immune system, also play a role in regulating T cell responses within the CNS [51]. Altering the function of peripheral DCs can influence the development of autoimmune neuroinflammation. Studies have shown that modifying DCs to enhance their production and secretion of IL-10 can facilitate the development of Tregs [52]. For instance, the loss of the R-Ras gene leads to the formation of DCs with reduced MHC-II expression levels, thereby promoting Treg proliferation and ultimately alleviating EAE symptoms [53]. Additionally, the JAK1 signaling pathway in DCs promotes peripheral tolerance in autoimmunity through a PD-L1-mediated mechanism of regulatory T cell induction [54]. In preclinical models of MS, retinoic acid (a metabolite of vitamin A) has been used to modulate DCs, enabling them to acquire the ability to induce Tregs and suppress Th17 cell polarization [55]. Consequently, DCs are not only capable of activating T cells and supporting their proliferation but can also alter the differentiation direction of T cells through the expression of surface molecules and secretion of cytokines [56]. Our study revealed that nebulized supernatant reprograms lung DCs toward a tolerogenic phenotype, exhibiting reduced expression of MHC-II, CD80, and CD40, decreased production of TNF-α and GM-CSF, and increased secretion of IL-10. Notably, intravenous supernatant delivery did not induce comparable DC changes in the lung, suggesting that localized pulmonary exposure is essential for this effect.

FOXO3 is a pivotal transcription factor that regulates the cell cycle and cell survival. It not only limits the production of key inflammatory cytokines by DCs but also controls T cell survival [19]. In studies of EAE, FOXO3 has been shown to regulate the differentiation of CD4^+^ T cells and modulate the severity of neuroinflammation, while promoting the differentiation of pathogenic T helper 1 cells through the induction of Eomes expression [57]. However, the role of FOXO3 in DCs during EAE has not been fully explored. This study found that nebulized BMSC supernatant may influence the secretion of inflammatory factors and the antigen-presenting function of DCs in the lung tissue of EAE mice by upregulating FOXO3 expression in DCs. This suggests that FOXO3 activation is a central mechanism by which nebulized supernatant exerts its immunomodulatory effects. To directly link tolDC induction to therapeutic efficacy, we performed adoptive transfer experiments. Transfer of BMSC supernatant–preconditioned BMDCs into EAE mice significantly delayed or even prevented disease onset and reduced clinical severity, while FOXO3 inhibition abrogated this benefit. This provides functional evidence that tolDCs generated by supernatant pretreatment are sufficient to confer protection against EAE, consistent with reports that tolDC-based therapies can suppress autoimmunity in preclinical models.

Finally, adoptive transfer of supernatant-preconditioned DCs modulated T cell differentiation in peripheral tissues, reducing Th1/Th17 polarization and increasing Treg proportions in the spleen and lymph nodes. These findings integrate well with our earlier observations on peripheral T cell modulation following nebulization and reinforce the idea that DC–T cell interactions in the periphery are critical for controlling CNS autoimmunity.

Taken together, our results support a mechanistic model in which nebulized BMSC supernatant is taken up primarily by lung DCs, induces a FOXO3-dependent tolerogenic phenotype, reprograms peripheral T cell responses toward immune tolerance, and ultimately reduces CNS inflammation and demyelination (see Fig. 7). This strategy leverages the lung–brain axis to deliver immunomodulation in a non-invasive manner, representing a promising acellular therapeutic approach for MS and potentially other autoimmune disorders.

However, this study has some limitations. First, while we confirmed FOXO3 involvement in DC modulation,, and literature has demonstrated an increase in PP2A expression in APC treated with exosomes, with PP2A being an effective upstream factor for the dephosphorylation of Foxo3 [58], downstream signaling pathways and epigenetic mechanisms warrant further exploration. Second, the biodistribution and long-term persistence of nebulized supernatant components remain unclear. BMSCs supernatant may inhibit the activation of the NLRP3 inflammasome and the release of associated cytokines, thereby alleviating inflammation, through its microRNAs, such as miR-197-3p, miR-223-3p, and miR-155 [59–61]. Although extensive literature has confirmed that BMDCs can home to the lung tissues of mice following adoptive transfer, and their in vitro phenotypic and functional changes are consistent with those of lung-resident DCs upon induction under identical conditions. Consequently, multiple therapeutic strategies including nanomedicines developed based on this finding have demonstrated promising translational potential [62, 63]. This can be attributed to the fact that DCs differentiate from bone marrow cells, whose differentiation process can be standardized in vitro; DCs throughout the body maintain a storage-circulation balance, with bone marrow cells serving as the core regulator of this equilibrium [64–67]. Furthermore, our parallel induction study on lung-resident DCs and BMDCs using magnetic-activated cell sorting (MACS)—a highly recognized technique—verified the reliability of previous research and provided a rational basis for our experiments, as evidenced by observed phenotypic alterations and upregulation of key transcription factors. However, as noted earlier, BMDCs cannot fully recapitulate the dynamic balance shifts and the integrated microenvironmental cues shaped by other cell populations in vivo. In-depth investigations would require advanced techniques such as in vivo dynamic imaging, fluorescent nanolabeling tracing, and high-resolution single-cell transcriptomics. Constrained by the limitations of our experimental platform and to ensure the reproducibility of the study, we anticipate conducting more comprehensive research in the future. Finally, translation to human MS will require optimization of nebulization parameters and validation in clinical settings.

## Supplementary Information

Supplementary Material 1.

## Data Availability

No datasets were generated or analysed during the current study.
