# Supplementary material for: Nebulized bone marrow-derived stem cell supernatant induces tolerogenic dendritic cells via upregulation of FOXO3 for EAE treatment
Source: J Neuroinflammation. 2026 Jan 14;23:59. doi: 10.1186/s12974-025-03690-2 (PMC12888473; doi:10.1186/s12974-025-03690-2)
Supplement: Supplementary file 1 — Supplementary Material 1. [file 12974_2025_3690_MOESM1_ESM.docx]

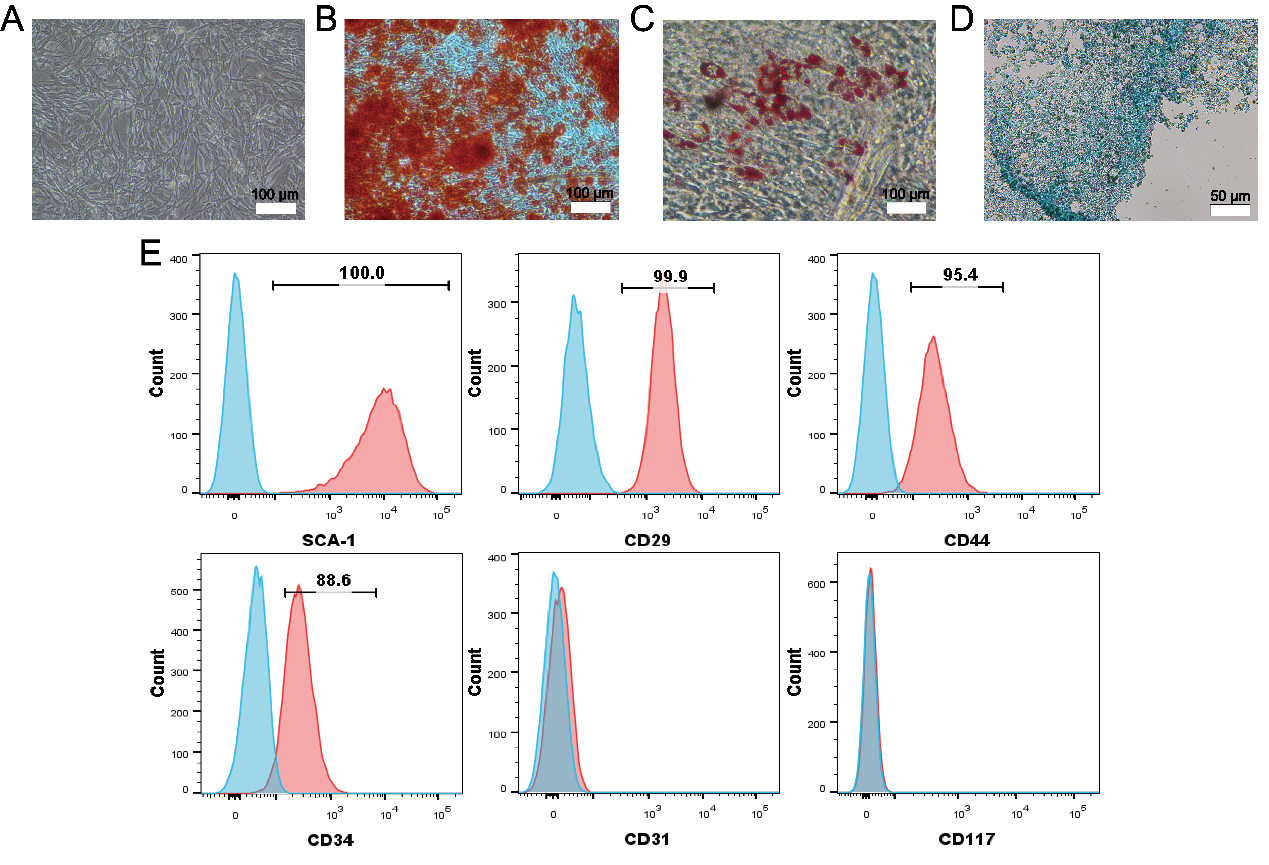


Fig. S1. Identification of primary BMSCs.

(A) The morphology of BMSC. Magnification 100×. (B) BMSC osteogenic differentiation. Magnification 100×. (C) BMSC alipogenic differentiation. Magnification 100×. (D) BMSC chondrogenic differentiation. Magnification 100×. (E) BMSC surface markers were identified by flow cytometry. CD29, CD44, CD34, SCA-1 are positive. CD117, CD31 are negative.


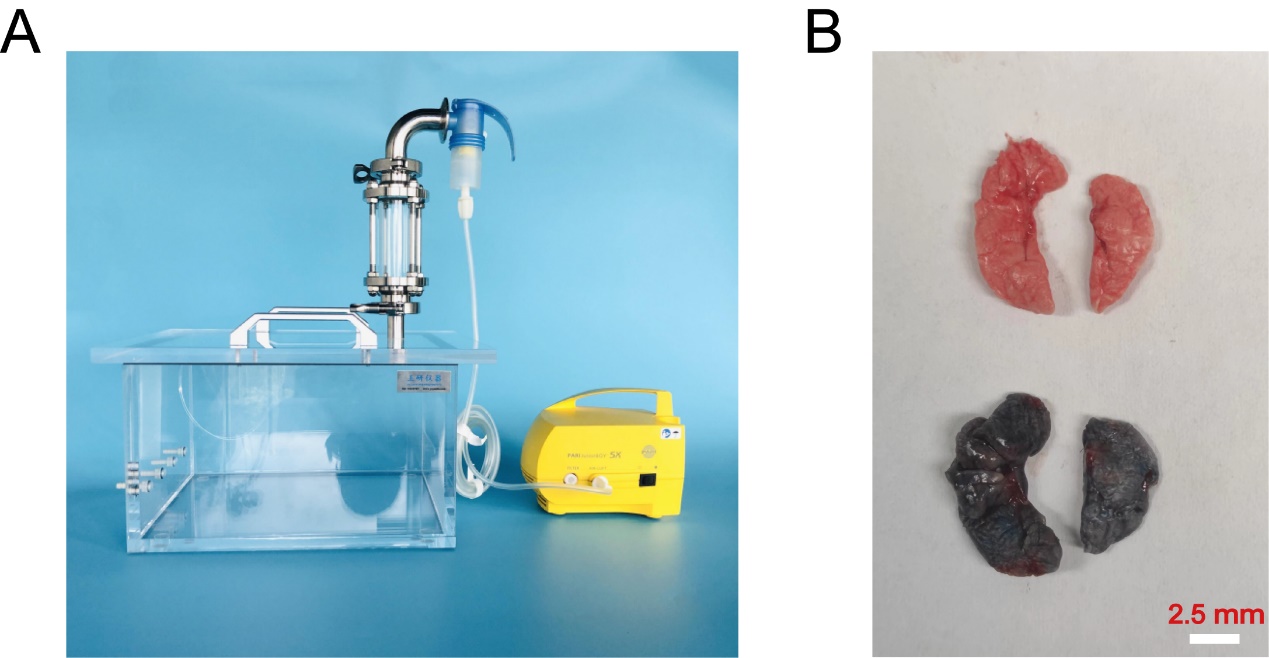


Fig. S2. (A) Assembly diagram of the atomization device and (B) the effect diagram of methylene blue atomization in mouse lung inhalation (below). Normal mouse lung(above).


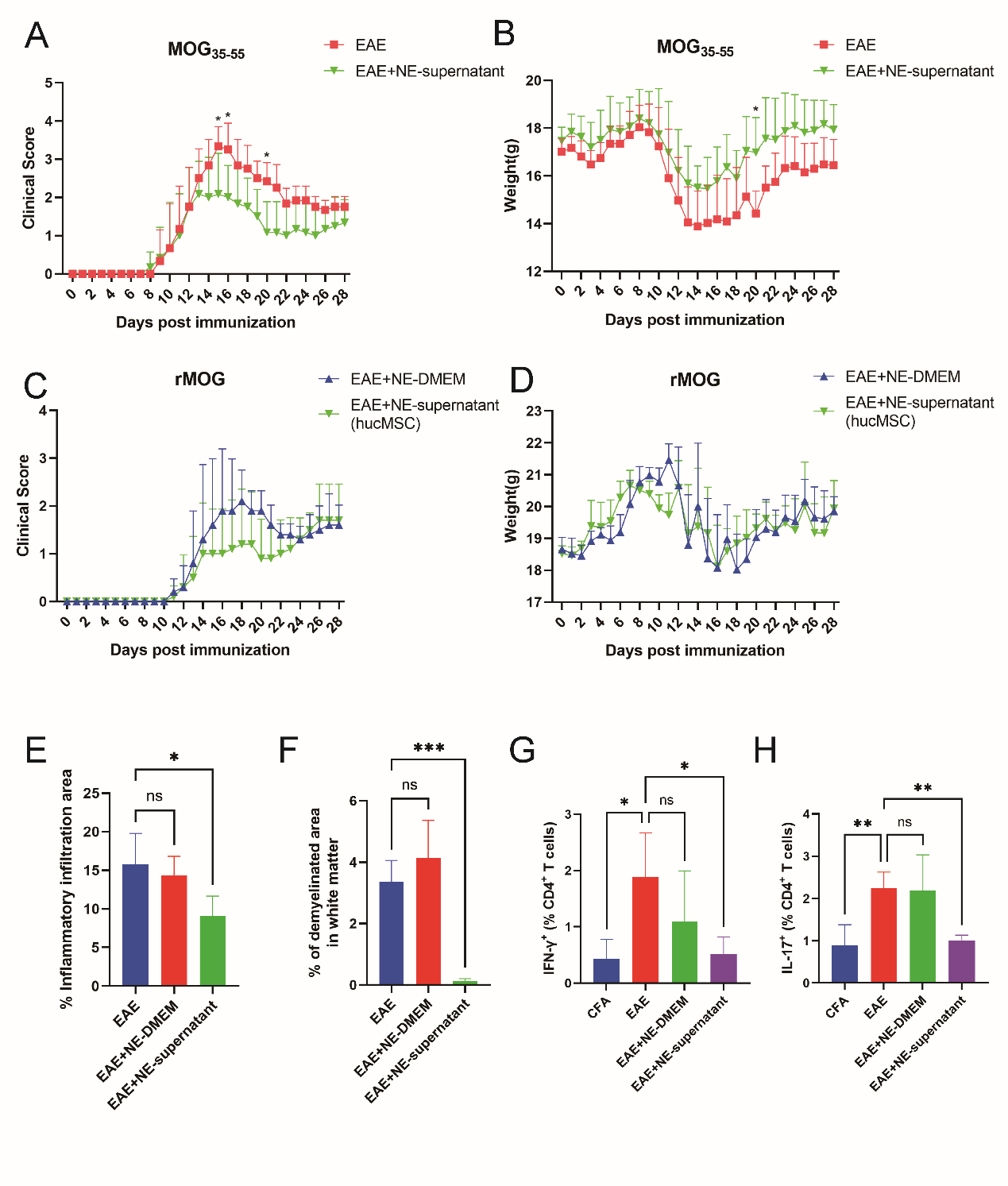


Fig. S3. Nebulized BMSC supernatant and hucMSC supernatant alleviate the severity of different Mog-induced EAE diseases.

(A) The clinical scores of Mog_35-55_-induced EAE and EAE+NE-supernatant group (n = 6). *Significant difference between EAE and EAE+NE-supernatant group. (B) The body weight evaluation of Mog_35-55_-induced EAE and EAE+NE-supernatant group (n = 6). (C) The clinical scores of rMog-induced EAE+NE-DMEM and EAE+NE-supernatant(hucMSC) group (n = 5). (D) The body weight evaluation of rMog-induced EAE+NE-DMEM and EAE+NE-supernatant(hucMSC) group (n = 5). (E) Percentage of inflammatory infiltration area in EAE, EAE + NE-DMEM and EAE + NE-supernatant groups. (F) Percentage of demyelination area in EAE, EAE + NE-DMEM and EAE + NE-supernatant groups. (n = 4). (G, H) The percentages of CD4+ T cells expressing IFN-γ and IL-17 in brain at the peak stage of disease (n = 5). (A-D) Data are expressed as Mean ± SD **p* < 0.05, *two-way ANOVA*. One-way ANOVA is used in E-H.


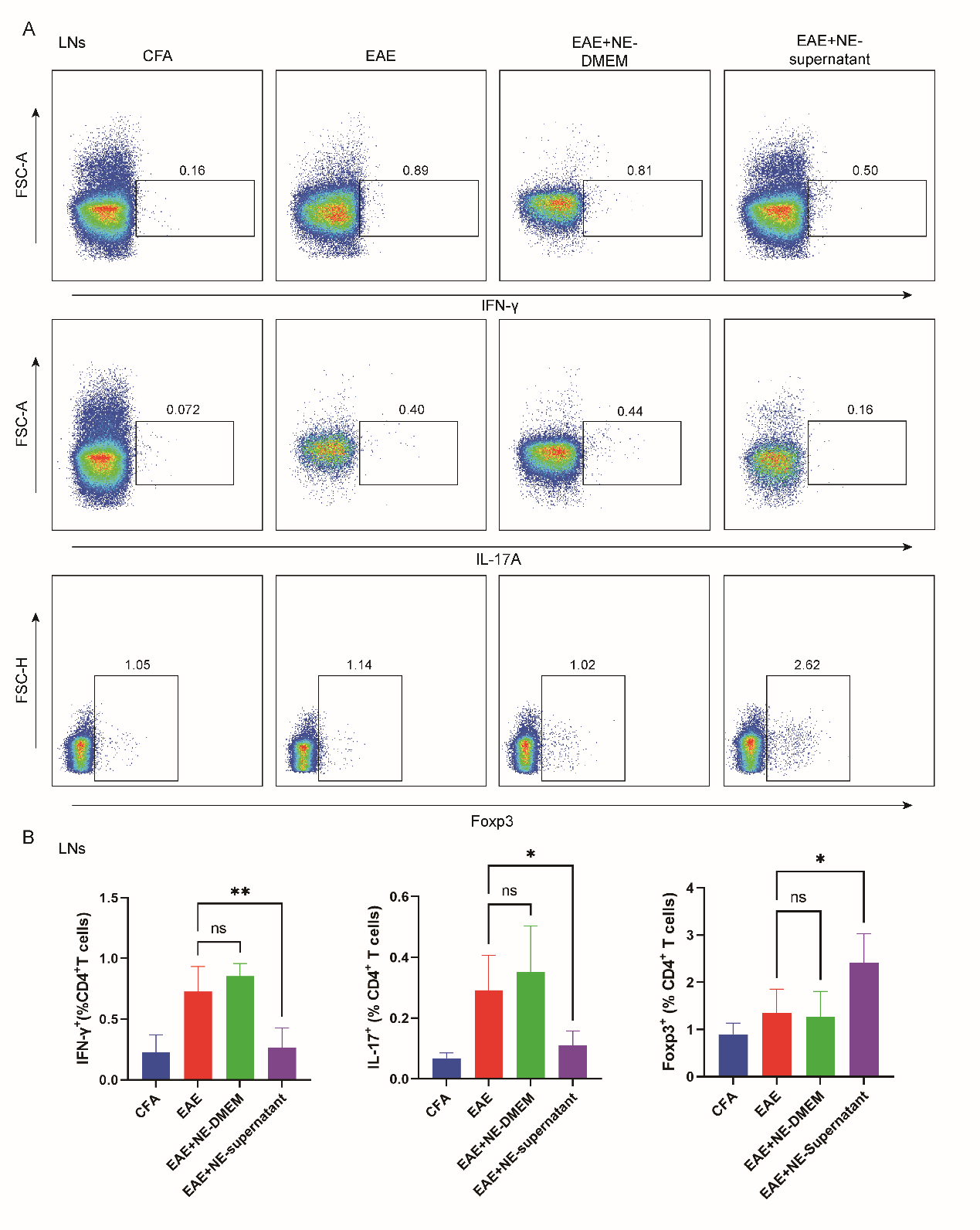


Fig. S4. Nebulized BMSC supernatant influences the differentiation of T cells in lymph node.

(A) Representative flow cytometry plots of CD4^+^ T cells expressing IFN-γ, IL-17 and Foxp3 in lymph node at the peak stage of disease. (B) The percentages of CD4^+^ T cells expressing IFN-γ, IL-17 and Foxp3 in lymph node at the peak stage of disease (n = 5). Data are expressed as Mean ± SEM, **p* < 0.05, ***p* < 0.01, ****p* < 0.001, *****p* < 0.0001, *one-way-ANOVA*.


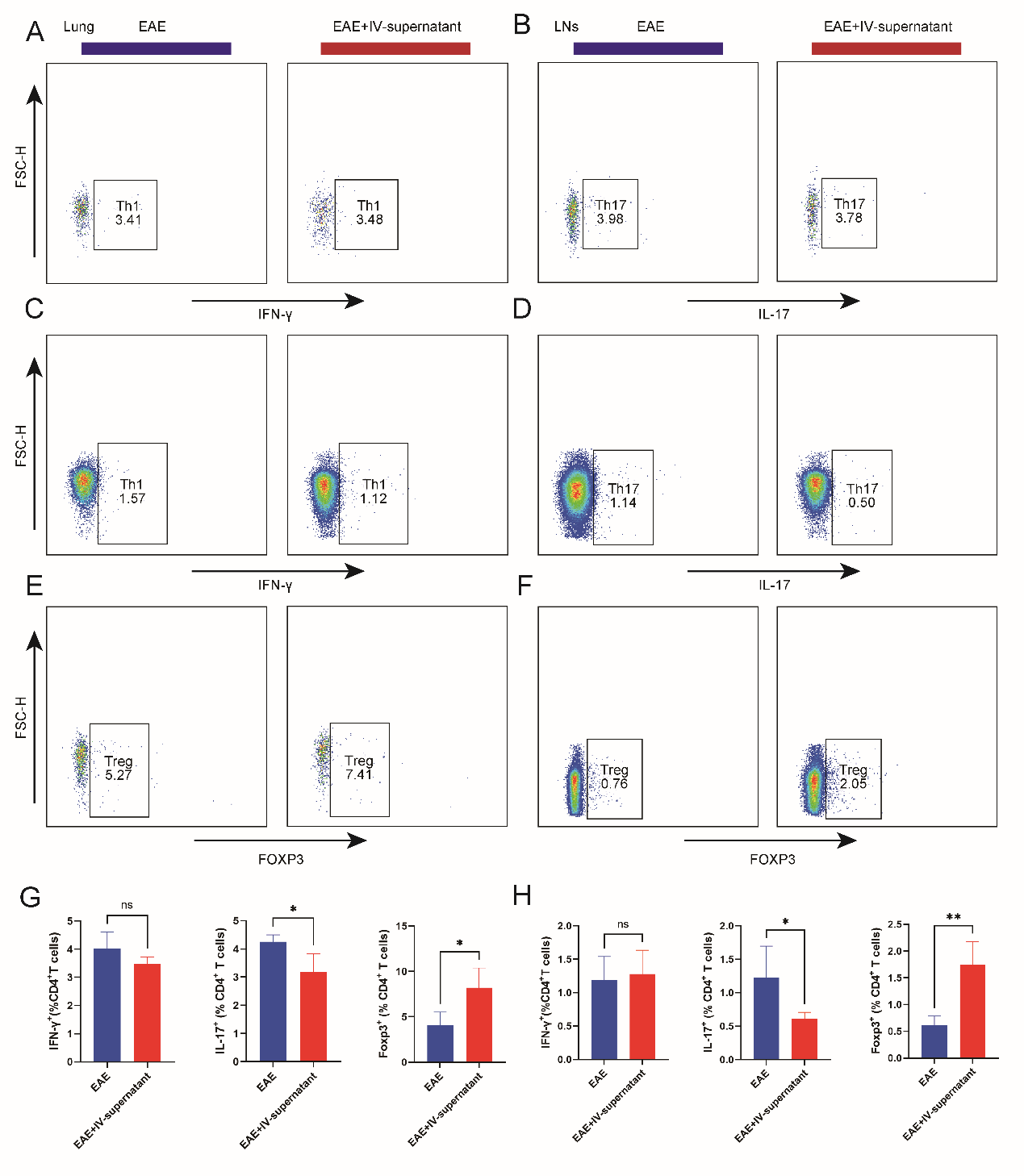


Fig. S5. T cell polarization in the lungs and lymph nodes of EAE and EAE + IV-supernatant groups.

(A-D) Changes in Th1 and Th17 cells in the lungs and lymph nodes of EAE and EAE + IV-supernatant groups. (E, F) Changes in Treg cells in the lungs and lymph nodes of EAE and EAE + IV-supernatant groups. (G) Changes in Th1, Th17 and Treg cells in the lungs of EAE and EAE + IV-supernatant groups (n = 4). (H) Changes in Th1, Th17 and Treg cells in the lymph nodes of EAE and EAE + IV-supernatant groups (n = 4). (G, H) Data are expressed as Mean ± SEM, **p* < 0.05, ***p* < 0.01, ****p* < 0.001, *t-test*.


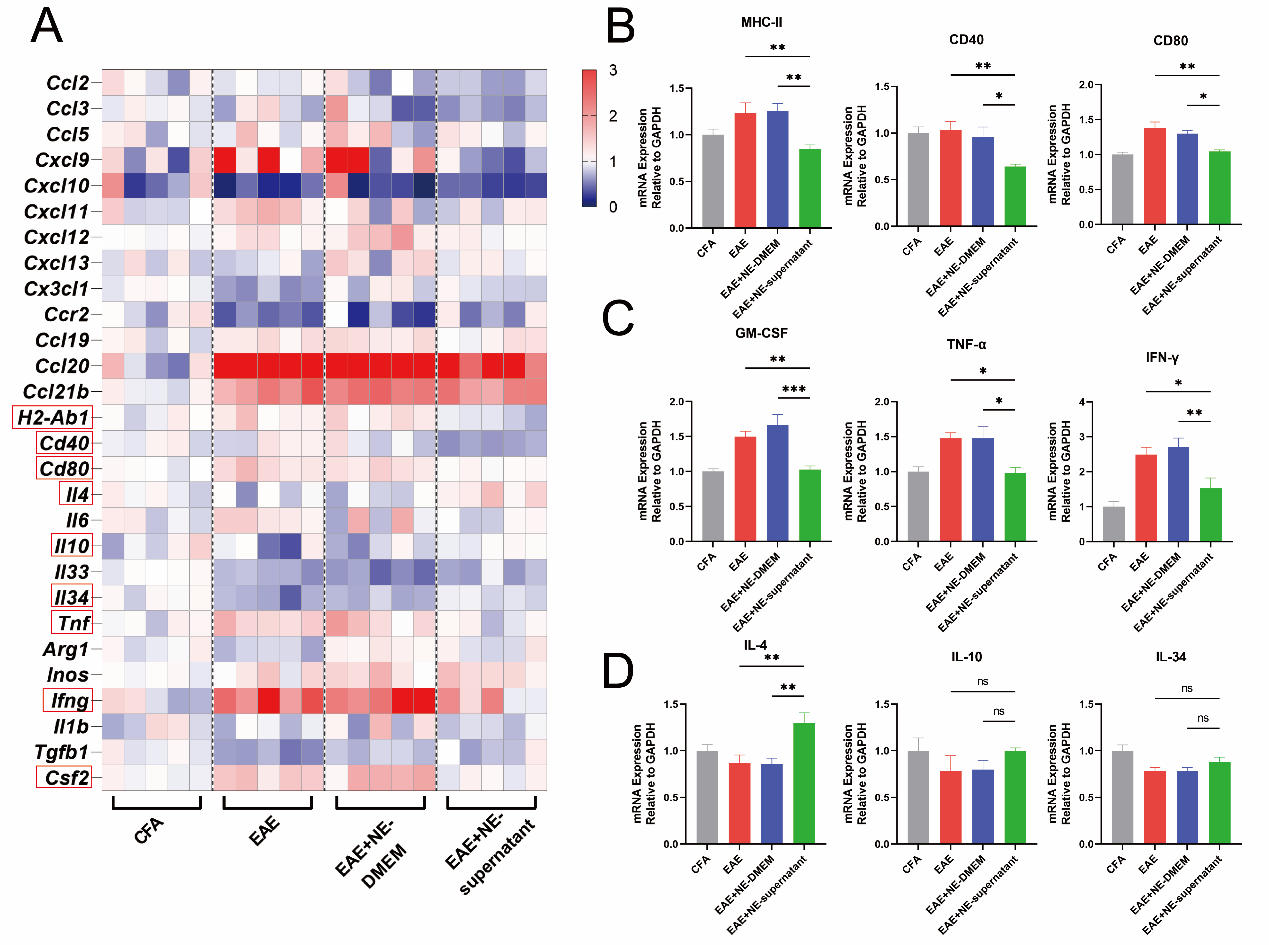


Fig. S6. Nebulized BMSC supernatant alleviates pulmonary tissue inflammation and remodels the pulmonary immune microenvironment in EAE mice.

(A) The heatmap illustrates the differential mRNA expression in lung tissue measured by qPCR. (B) The expression of antigen-presenting related mRNA (*H2-Ab1*, *Cd40* and *Cd80*) in lung tissue (n = 5). (C) The expression of pro-inflammatory related mRNA (*Csf2*, *Tnf* and *Ifng*) in lung tissue (n = 5). (D) The expression of anti-inflammatory related mRNA (*Il4*, *Il10* and *Il33*) in lung tissue (n = 5). (B-D) Data are expressed as Mean ± SEM, **p* < 0.05, ***p* < 0.01, ****p* < 0.001, *one-way-ANOVA*.


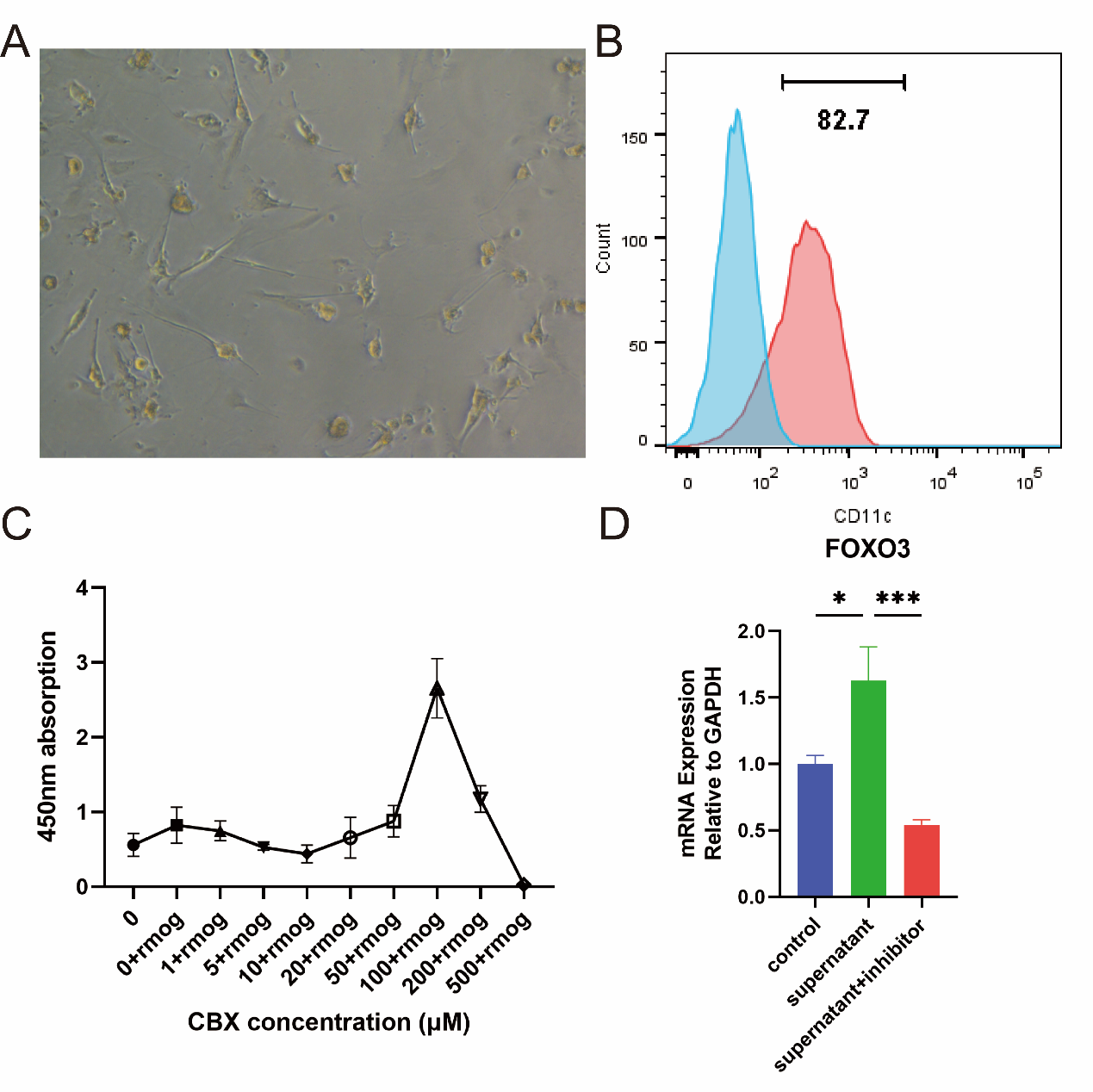


Fig. S7. Extraction and Identification of BMDCs.

(A) Immature dendritic cells exhibit a limited number of dendritic protrusions and show less cell clustering. Magnification 100×. (B) Immature BMDCs surface marker (CD11c) was identified by flow cytometry. (C) The cell viability of dendritic cells co-cultured with CBX was measured at 450 nm using the CCK8 assay. At a concentration of 100 µM CBX, the viability of DC cells was found to be optimal. (D) The mRNA expression level of *foxo3* in BMDC cultured in vitro was assessed (n = 5). Data are expressed as Mean ± SEM, **p* < 0.05, ****p* < 0.001, *one-way-ANOVA*.


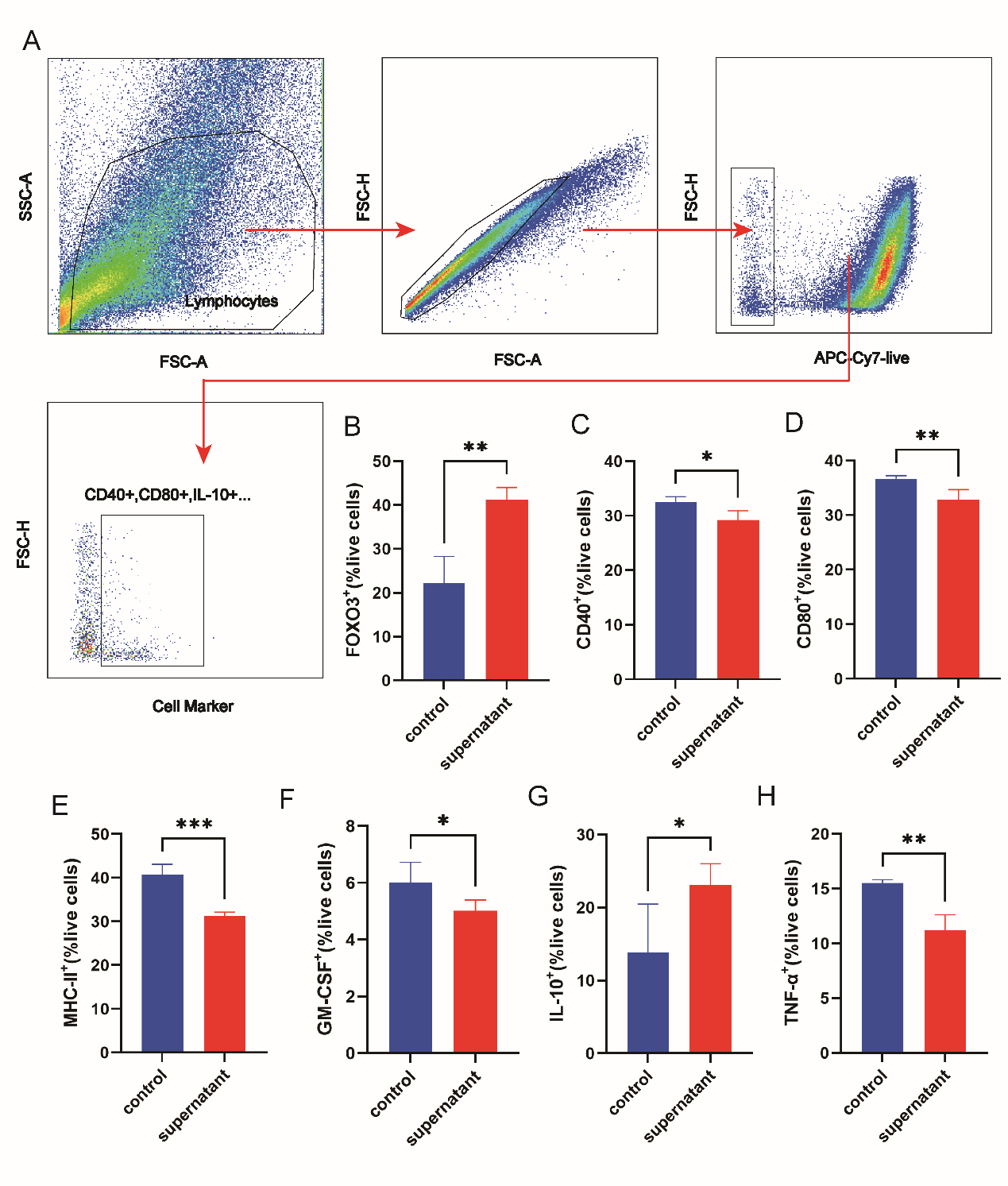


Fig. S8. Changes in lung tissue DCs induced by BMSC supernatant.

(A) Flow Cytometry Analysis Protocol for Identifying the Related Characteristics of Lung tissue DCs. (B-H) Alterations in the phenotypic characteristics of lung tissue DCs in control and supernatant groups (n = 4). (B-H) Data are expressed as Mean ± SEM, **p < 0.05, **p < 0.01, ***p < 0.001, t-test.*


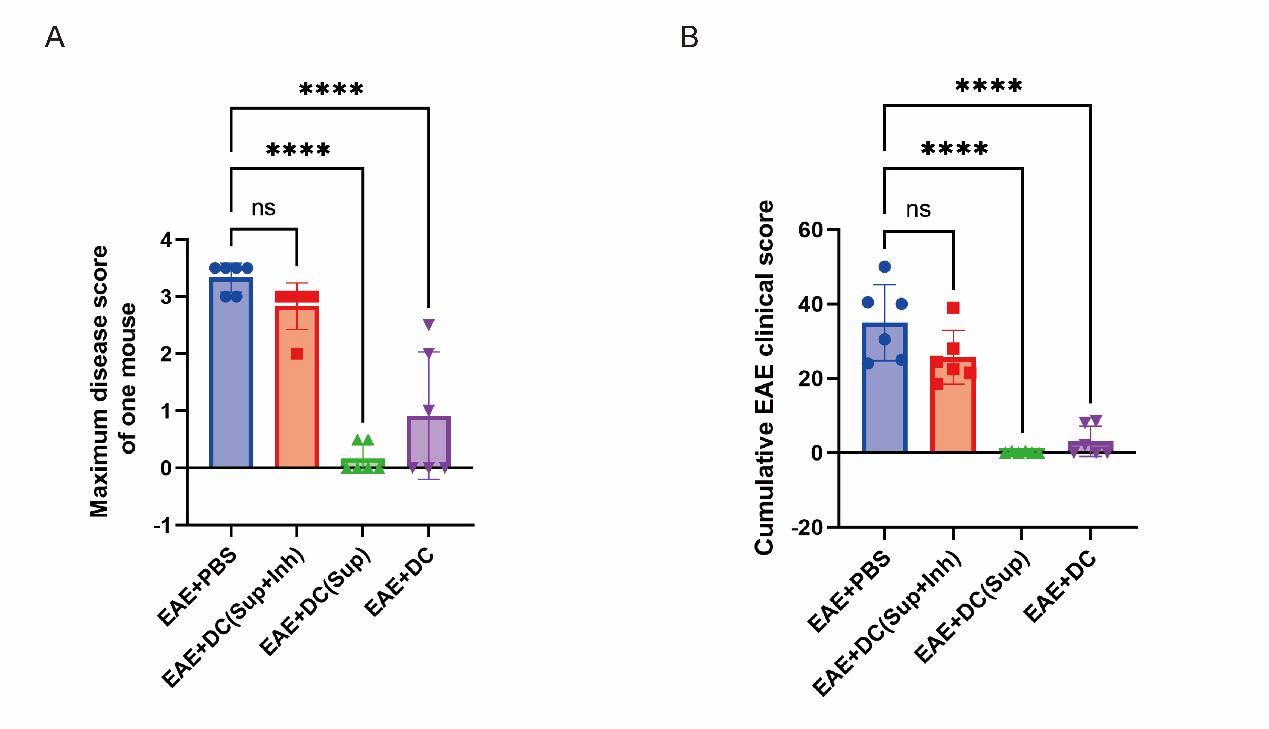


Fig. S9. The maximum individual clinical score and cumulative individual clinical score of mice in the EAE+PBS group, EAE+DC (Sup+Inh) group, EAE+DC (Sup) group and EAE+DC group.

(A) The maximum individual clinical score of mice in the EAE+PBS group, EAE+DC (Sup+Inh) group, EAE+DC (Sup) group and EAE+DC group (n = 6). (B) The cumulative individual clinical score of mice in the EAE+PBS group, EAE+DC (Sup+Inh) group, EAE+DC (Sup) group and EAE+DC group (n = 6). Data are expressed as Mean ± SEM, **p* < 0.05, ***p* < 0.01, ****p* < 0.001, *****p* < 0.0001, *one-way-ANOVA*.


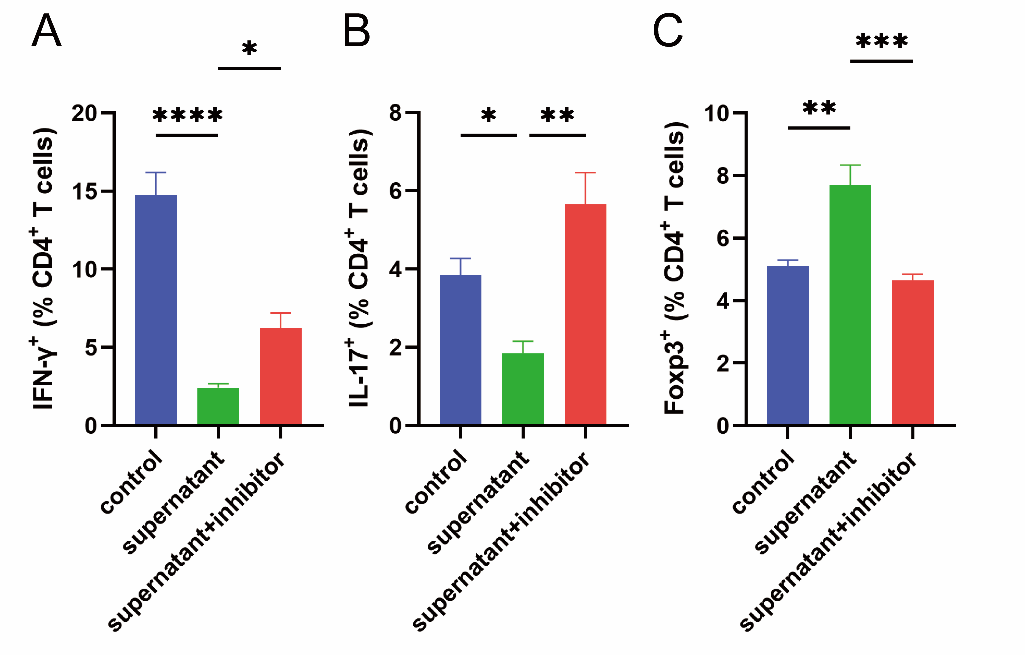


Fig. S10. The pre-treated BMDC co-cultured with splenic cells inhibited the differentiation of T cells into Th1 and Th17 subsets while promoting the differentiation of Treg cells.

(A) The percentage of T cells expressing IFN-γ after co-culturing preprocessed BMDCs with splenic cells (n = 5). (B) The percentage of T cells expressing IL-17 after co-culturing preprocessed BMDCs with splenic cells (n = 5). (C) The percentage of T cells expressing Foxp3 after co-culturing preprocessed BMDCs with splenic cells (n = 5). Data are expressed as Mean ± SEM, **p* < 0.05, ***p* < 0.01, ****p* < 0.001, *****p* < 0.0001, *one-way-ANOVA*.
